# Microtubule-actin crosslinking factor 1 (Macf1) domain function in Balbiani body dissociation and nuclear positioning

**DOI:** 10.1371/journal.pgen.1006983

**Published:** 2017-09-07

**Authors:** Matias Escobar-Aguirre, Hong Zhang, Allison Jamieson-Lucy, Mary C. Mullins

**Affiliations:** Department of Cell and Developmental Biology, University of Pennsylvania Perelman School of Medicine, 1152 BRBII/III, 421 Curie Blvd., Philadelphia, Pennsylvania, United States of America; Stanford University School of Medicine, UNITED STATES

## Abstract

Animal-vegetal (AV) polarity of most vertebrate eggs is established during early oogenesis through the formation and disassembly of the Balbiani Body (Bb). The Bb is a structure conserved from insects to humans that appears as a large granule, similar to a mRNP granule composed of mRNA and proteins, that in addition contains mitochondria, ER and Golgi. The components of the Bb, which have amyloid-like properties, include germ cell and axis determinants of the embryo that are anchored to the vegetal cortex upon Bb disassembly. Our lab discovered in zebrafish the only gene known to function in Bb disassembly, *microtubule-actin crosslinking factor 1a* (*macf1a*). Macf1 is a conserved, giant multi-domain cytoskeletal linker protein that can interact with microtubules (MTs), actin filaments (AF), and intermediate filaments (IF). In *macf1a* mutant oocytes the Bb fails to dissociate, the nucleus is acentric, and AV polarity of the oocyte and egg fails to form. The cytoskeleton-dependent mechanism by which Macf1a regulates Bb mRNP granule dissociation was unknown. We found that disruption of AFs phenocopies the *macf1a* mutant phenotype, while MT disruption does not. We determined that cytokeratins (CK), a type of IF, are enriched in the Bb. We found that Macf1a localizes to the Bb, indicating a direct function in regulating its dissociation. We thus tested if Macf1a functions via its actin binding domain (ABD) and plectin repeat domain (PRD) to integrate cortical actin and Bb CK, respectively, to mediate Bb dissociation at the oocyte cortex. We developed a CRISPR/Cas9 approach to delete the exons encoding these domains from the *macf1a* endogenous locus, while maintaining the open reading frame. Our analysis shows that Macf1a functions via its ABD to mediate Bb granule dissociation and nuclear positioning, while the PRD is dispensable. We propose that Macf1a does not function via its canonical mechanism of linking two cytoskeletal systems together in dissociating the Bb. Instead our results suggest that Macf1a functions by linking one cytoskeletal system, cortical actin, to another structure, the Bb, where Macf1a is localized. Through this novel linking process, it dissociates the Bb at the oocyte cortex, thus specifying the AV axis of the oocyte and future egg. To our knowledge, this is also the first study to use genome editing to unravel the module-dependent function of a cytoskeletal linker.

## Author summary

The totipotent egg of most vertebrates is polarized in a so called animal-vegetal axis that is essential to early embryonic development. The animal-vegetal axis is established in the early oocyte by the dissociation of the Balbiani Body (Bb). The Bb is a large RNA-protein granule, conserved from insects to mammals, that forms next to the oocyte nucleus and dissociates later at the oocyte cortex. Importantly, Bb dissociation at the oocyte cortex defines the future vegetal pole of the egg. Macf1a, a cytolinker, is the only factor known to regulate Bb dissociation. However, how the giant Macf1a protein with multiple functional domains can interact with the cytoskeleton to regulate Bb disassembly is unknown. Here, we unravel Macf1a function via interrogating, for the first time, individual *macf1a*-encoded domains of the gene in its normal chromosomal location for their requirement in Bb dissociation and ultimately in egg polarity establishment. The method presented here is applicable to other cytolinkers involved in human disease.

## Introduction

Cellular polarity organizes the intracellular space into cytoplasmic domains that mediate cellular functions across diverse cell types. For instance, oocytes are polarized in many species with the formation of the Balbiani Body (Bb) (also called the mitochondrial cloud in Xenopus) adjacent to the nucleus. The Bb is a large granule conserved from insects to mammals that tightly aggregates RNAs, proteins, ER, and mitochondria. The Bb granule is a non-membrane bound compartment that isolates its content from the cytoplasm. There is evidence in zebrafish and Xenopus stage I oocytes that the Bb forms through the assembly of Bucky ball amyloid-like fibers that capture Bb components and give rise to the large Bb granule [1–3]. Later, by the end of stage I (stage II in Xenopus), the Bb dissociates at the oocyte cortex and its components become docked at the now defined oocyte vegetal pole. This establishes the animal-vegetal (AV) axis of the oocyte and future egg, which in turn defines the anterior-posterior axis of the embryo [4]. Hence, elucidating the mechanism of Bb disassembly is relevant to understanding two conserved and linked processes; the establishment of cell polarity and the disassembly of an amyloid-like structure such as the large Bb granule.

The two proteins known to be necessary for Bb function in zebrafish, Bucky ball (Buc) and Microtubule-actin crosslinking factor 1a (Macf1a), were discovered via a zebrafish maternal-effect mutant screen in our lab [2, 5, 6]. In eggs from *buc* or *macf1a* mutant females, the cytoplasm fails to segregate to form the blastodisc at the animal pole, and instead is radially distributed around the yolk [6]. Lacking AV polarity, development aborts shortly thereafter [6]. During early stage I of zebrafish oogenesis, Bucky ball is required for Bb formation, and the *Xenopus* Buc ortholog, Xvelo, is the most abundant protein in the frog Bb [1, 3]. *buc* mutant oocytes lack a Bb and RNAs normally carried within the Bb are dispersed throughout the cytoplasm and never localize to the vegetal pole [2, 3, 7]. Xvelo self-aggregates *in vitro* and *in vivo* forming a matrix of amyloid-like fibers that may entrap mitochondria to create the Bb [1, 8]. These amyloid-like aggregates are very stable and difficult to disrupt [1]. However, the Bb naturally disassembles by the end of stage I of oogenesis. Macf1a is the only known functional player in this process. In *macf1a* mutant oocytes the Bb forms and accumulates RNA normally, however, the Bb becomes enlarged and never disassembles [5]. All *macf1a* mutant oocytes also develop an acentric nucleus phenotype beginning at mid-stage 1 of oogenesis [5]. It is unknown if this phenotype is functionally linked to AV polarity and Bb disassembly. Understanding the role of Macf1a in Bb dissociation could provide insight into the dissociation of similar amyloid-like aggregations in pathological conditions.

Macf1 is a conserved, giant cytolinker that can interact with all cytoskeleton components: microtubules (MTs), actin filaments (AF) and intermediate filaments (IF). Macf1 is modular in that distinct domains can interact with each of these cytoskeleton components, and these distinct domains are expressed in multiple isoforms [9–11]. Macf1 functions in a variety of tissues and processes in different species, all integrating the cytoskeleton in cellular functions. In mice, Macf1 is essential for early development and mutant embryos die during gastrulation [12]. In mammalian cells, Macf1 acts as a plus (+) tip MT-binding protein and mediates MT and actin integration at the cell cortex [10, 13]. Keratinocytes require Macf1 for cell migration, where Macf1 integrates MTs and actin cables to maintain focal adhesions [13, 14]. Similarly, using the gene trap line Gt*(macf1a-citrine)*^*ct68a*^ in zebrafish, *Antonellis et al* [14] showed that Macf1a-Citrine fusion protein likely functions in connecting MTs to actin in hair cells and participates in apical-basal polarity. In invertebrates, the *macf1* orthologs, *shot* (fly) and *VAB-10* (worm), have diverse functions in axon targeting, nuclear migration, epidermal attachment, and germ cell maintenance [15–23].

How Macf1a interacts with the cytoskeleton to disassemble the Bb and establish oocyte polarity remains undetermined [8, 24, 25]. Technical constraints have restricted the study of *macf1a*, since it is a large gene spanning ~300 kb of the zebrafish genome, with the longest predicted ORF of ~25 kb (NCBI: XP_001920094.1). Using transgenes for such large transcripts is difficult and subject to variable expression due to heterogeneous insertion sites. Thus, to unambiguously determine how Macf1a acts in AV polarity establishment, we targeted the *macf1a* endogenous gene to address Macf1a domain function in its normal physiological context.

Here we investigated the localization and function of Macf1a and the cytoskeleton in regulating the Bb and oocyte nucleus positioning. We found that Macf1a and cytokeratins (a type of IF) localize to the Bb, and that Macf1a associates with actin at the cortex upon Bb disassembly. Disruption of cortical actin in late stage I oocytes causes detachment of Bb components from the cortex, partially phenocopying the *macf1a* mutant. In contrast, disruption of MTs does not affect the Bb or nuclear positioning. Based on these results, we tested the hypothesis that Macf1a functions via its ABD and/or PRD (IF binding domain) to regulate Bb disassembly at the cortex. To test this, we used CRISPR/Cas9 genome editing technology to delete these domains by targeting the *macf1a* endogenous gene. This method harnesses the modular structure of the Macf1 cytoskeleton-binding domains to specifically interrogate single Macf1a domain functions in Bb disassembly and nucleus positioning. Our results reveal that the Macf1a ABD is essential for Bb disassembly and correct nuclear positioning. Surprisingly, we found that the Macf1a PRD domain is dispensable for both of these processes. To our knowledge, this is the first study to use genome editing to precisely target the module-dependent function of a cytoskeletal linker.

## Results

**Fig 1:**
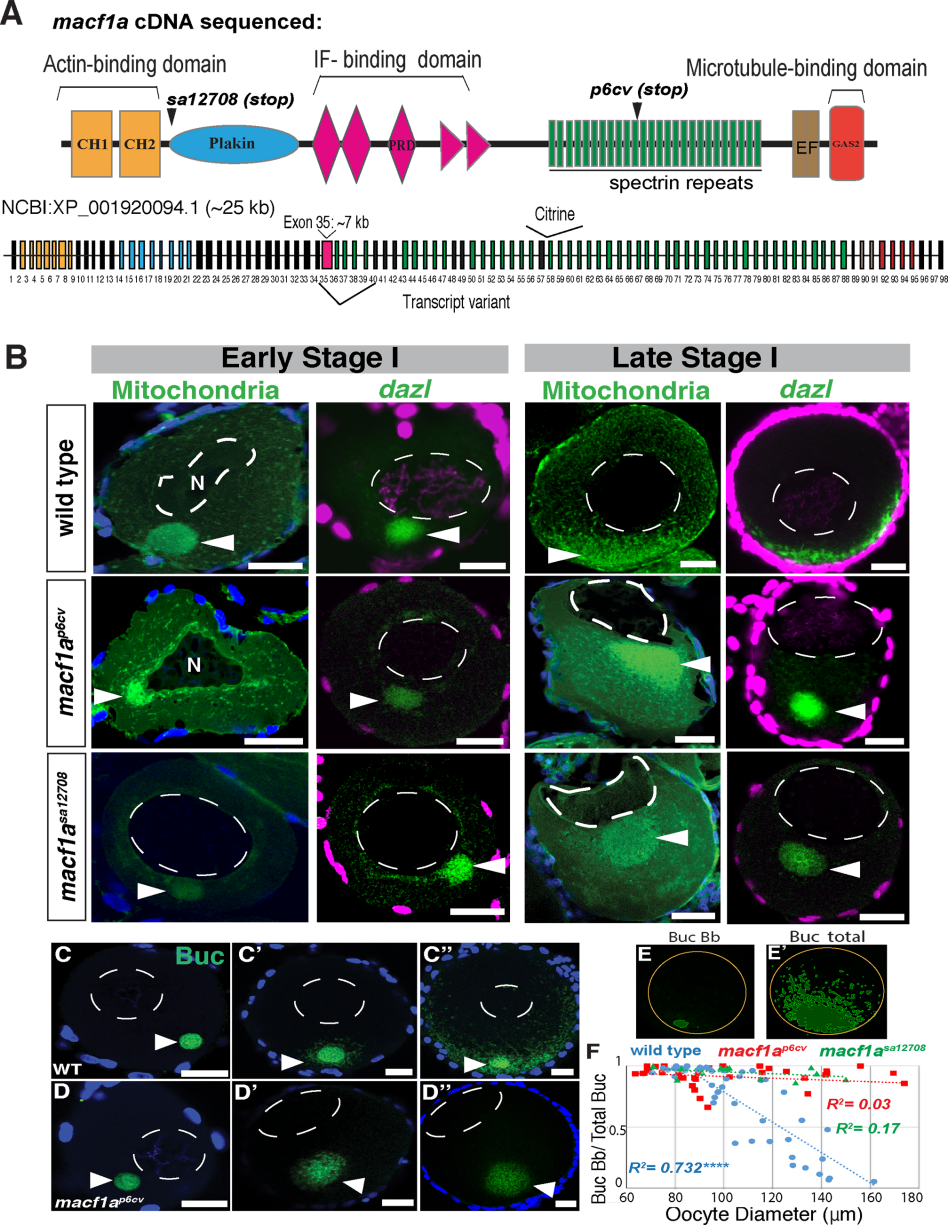
Macf1a is essential in relocalizing Buc from the Bb to the oocyte cortex. A) *macf1a* ovary cDNA from zebrafish was sequenced to determine the Macf1a domains that are expressed. CH = calponin-homology domain (yellow); PRD = Plectin repeat domain (pink); EF = Ca^+2^ binding domain (brown); Gas2: MT binding domain (red); IF = Intermediate filament. The exons are numbered and color-coded according to the domains. The premature stop codon locations in mutant alleles, *macf1a*^*sa12708*^ and *macf1a*^*p6cv*^, are shown. B**)** DiOC6 staining (mitochondria, green) and *dazl* in situ (green) in early and late stage I oocytes, labeling in WT the Bb prior to disassembly (early stage I) and the disassembled Bb at the cortex (late stage I). *macf1a*^*p6cv*^ and *macf1a*^*sa12708*^ mutant oocytes display a normal Bb in early stage I, but in late stage I the Bb enlarges and never disassembles. In addition, *macf1a* mutants show an acentric nuclear position compared to WT. DiOC_6_: N≥5 ovaries, >30 WT, *macf1a*^*p6cv*^, and *macf1a*^*sa12708*^ oocytes. *dazl* in situ: N = 5 ovaries, >30 WT, 25 *macf1a^p6cv^* and 35 *macf1a*^*sa12708*^ oocytes. C-D) Buc staining (green) to visualize Bb disassembly in stage I oocytes in WT and *macf1a* mutants (C-D, images correspond to different oocytes). E) Quantification method for Bb disassembly during stage I. The two images (E-E’) correspond to a Z-stack from C” where green represents the areas of Buc Bb and Buc total of oocytes that were segmented according to Buc signal intensity (see methods). The yellow circle marks the oocyte perimeter, which was used to estimate the oocyte diameter. F) Bb disassembly versus oocyte size (60 μm diameter early stage I to 160 μm late stage I) in WT, *macf1a*^*p6cv*^ and *macf1a*^*sa12708*^ mutants. Buc Bb/Total Buc (a quantitative measure of Bb disassembly) decreases as oocyte diameter (μm) increases, and this correlation between X and Y values is significant in WT (R^2^ = 0.732, correlation coefficient. **** P value < 0.0001), but not in *macf1a*^*p6cv*^ (R^2^ = 0.03, P value = 0.2377) and *macf1a*^*sa12708*^ (R^2^ = 0.17, P value = 0.1764). N≥ 3 ovaries, WT and mutant oocytes. DAPI staining labels DNA (blue or magenta) and marks follicle cells. Images are single optical sections, except for E-E’. Arrowheads indicate Bb and N the nucleus. Scale bar: 20 μm. All images are representative from at least 3 experiments.

In the zebrafish genome two paralogs of *macf1* are present, *macf1a* and *macf1b*. *macf1a* is expressed during oogenesis and a mutation in *macf1a* causes the AV oocyte and egg polarity phenotype [5] and is the focus of this study. The largest predicted *macf1a* open reading frame (ORF) in zebrafish is very large: ~25 kb, encoding a protein of ~8,000 amino acids (NCBI: XP_001920094.1). To determine the domain composition of *macf1a* transcripts in oogenesis, we sequenced *macf1a* cDNA from the ovary. We detected all predicted cytoskeleton interaction domains and other conserved domains of Macf1 in *macf1a* ovary cDNA, including the N-terminal ABD followed by the Plakin domain, 29 Spectrin repeats, and at the C-terminus, two EF-hands (Ca^2+^ binding motif) and a GAS2 microtubule binding domain (MTBD) for MT interaction (Fig 1A).

The predicted ORF (NCBI: XP_001920094.1) of the *macf1a* gene also contains the PRD domain between the Plakin domain and the Spectrin repeats (Fig 1A), similar to the *macf1b* isoform in the mouse [11]. Like the mouse *macf1b* isoform, the PRD domain in zebrafish is contained in one large exon (exon 35) of ~7 kb. Since it is challenging to isolate 7 kb cDNA products without amplifying genomic DNA of this large exon, we instead used primers in flanking exons 34 and 36 with exon 35 primers to amplify part of exon 35 from ovary cDNA (Fig 1A). We sequenced ~2 kb from each side of the PRD showing that it is expressed during oogenesis. We also sequenced a cDNA that lacks the PRD and 13 Spectrin repeats; in this cDNA exon 34 is joined to exon 40, as well as exon 56 to 76, suggesting that alternative splicing generates a transcript lacking exons 35–39, and 57–75. We did not confirm splice forms lacking exons 57–75 in ovary cDNA, but did confirm alternate splice forms either containing or lacking exons 35–39 (Fig 1A, transcript variant). As also reported in the mouse [9, 11], this indicates that more than one *macf1a* isoform is generated in the zebrafish ovary. Altogether, we detected all Macf1a functional domains in *macf1a* ovary cDNA, including the PRD, encompassing all predicted exons (Fig 1A) of the longest *macf1a* ORF.

We previously isolated the *macf1a*^*p6cv*^ allele [6], which is a 31 bp deletion causing a frameshift in the ORF [5]. In this allele, the C-terminal half of the protein is predicted to be truncated at amino acid 5315 of the longest predicted zebrafish Macf1a isoform (>8000 amino acids) by a premature stop codon (Fig 1A, *p6cv* (stop)) [5, 6] (NCBI: XP_001920094.1). We obtained a second *macf1a* mutant allele from the Sanger Zebrafish Mutation Project (http://www.sanger.ac.uk/science/collaboration/zebrafish-mutation-project), *macf1a*^*sa12708*^, which shows an indistinguishable phenotype to *macf1a*^*p6cv*^ mutants, including Bb enlargement, failure of Bb disassembly, and acentric positioning of the nucleus, all of which are fully penetrant like in the *macf1a*^*p6cv*^ allele [5] (Fig 1B). The new allele is a nonsense point mutation affecting codon 553 near the N-terminus of Macf1a (Fig 1A, *sa12708* (stop)). These two alleles contain premature stop codons at very different locations in the ORF and yet display the same defects, which provides strong evidence that both are strong loss-of-function or null alleles.

### Macf1a is essential in Buc relocalization from Bb to oocyte cortex

The Bb progresses during stage IB of oogenesis from its initial location adjacent to the nucleus, to the oocyte cortex by late stage IB where it disassembles. To characterize this process in *macf1a* mutants, we followed protein and RNA markers of the Bb in mutant and wild type (WT) stage IB oocytes ranging from ~50 to 140 μm in diameter [26]. The Bb markers we selected were Buc, the only protein known to be required for Bb formation in vertebrates, and *dazl*, an mRNA component of the germ plasm (Fig 1B–1D). To examine *dazl* RNA localization, we used the highly sensitive hybridization chain reaction (HCR) method that allows detection of low RNA concentrations with minimal background [27]. We observed that Buc is recruited to the Bb in *macf1a* mutant oocytes (Fig 1D’ and 1D”), similar to *dazl* and other previously examined Bb components (Fig 1B) [5, 28]. Unlike in WT, in *macf1a* mutants Buc and *dazl* fail to localize to the vegetal cortex and instead remain in a persistent and enlarged Bb (Fig 1B–1D”).

We used the Buc immunofluorescence localization pattern to quantify the progression of Bb disassembly during stage I of oogenesis in WT and *macf1a* mutant oocytes (Fig 1C and 1D”). In late stage IB WT oocytes, as the Bb disassembles at the cortex, Buc dissociates from the Bb and localizes to the vegetal cortex (Fig 1C and 1C”). To quantitatively evaluate Bb disassembly, we identified the Bb by Buc immunostaining, then compared the signal intensity of Buc within the Bb (Fig 1E) versus outside the Bb (Fig 1E’) during disassembly (see methods). We measured the total Buc immunofluorescence area versus Buc localized to the Bb to estimate a Bb disassembly ratio (Bb Buc/Buc total), along with measuring the oocyte diameter throughout stage I (Fig 1F). In 60 micron (μm) early stage IB oocytes when the mature, compact Bb has formed (Fig 1C) (Elkouby et al, 2016), the Buc disassembly ratio is ~1. This ratio decreases to ~0 towards the end of stage I when the Bb disassembles and Buc is unloaded at the cortex (Fig 1F).

Using this method, we found that the Bb begins to dissociate at the vegetal pole in WT oocytes that were 95 to 110 μm in diameter, and reached the midway point (0.6 to 0.4 Buc disassembly ratio) in oocytes 115 to 125 μm in diameter (Fig 1F). In the largest WT stage IB oocytes (135 to 160 μm in diameter) the disassembly ratio was ~0.15. In contrast, the Bb disassembly ratio in *macf1a*^*p6cv*^ *and macf1*^*sa12708*^ mutant oocytes of a similar diameter range did not appreciably decrease below 1 (Fig 1F, red squares and green triangles, respectively). These results demonstrate that Macf1a is essential to dissociate the Bb granule and relocalize Buc, the essential Bb-forming protein, from the Bb to the cortex to establish AV oocyte polarity. Furthermore, this analysis indicates that the Bb does not dissociate during a short window of time, but rather disassembles over a period of oocyte growth that corresponds to an almost 2-fold increase in volume (from <120 μm to 145 μm diameter).

### Macf1a functions independently in Bb disassembly and nucleus positioning

**Fig 2:**
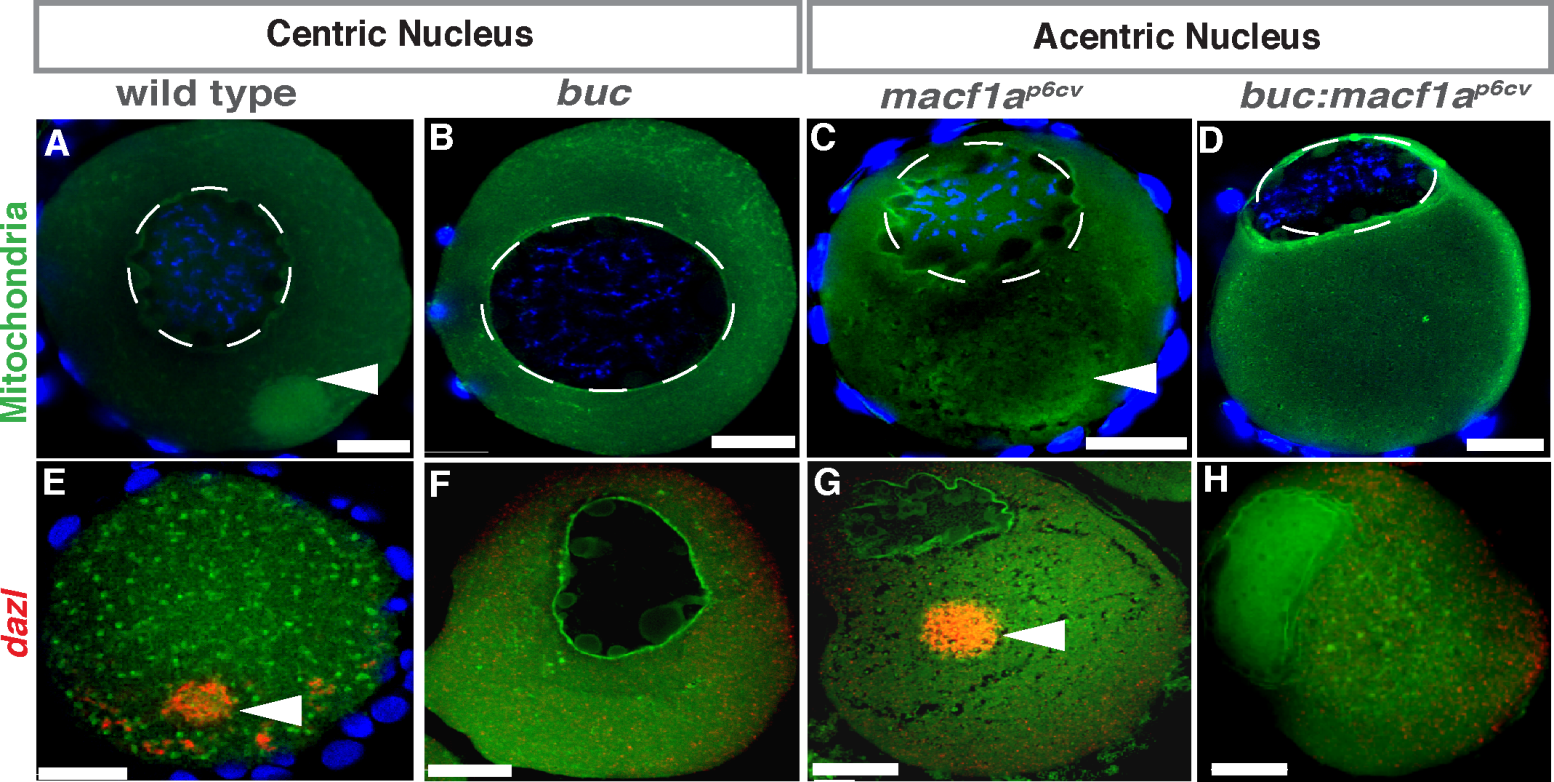
Epistasis of *bucky ball* and *macf1a* in nuclear positioning. A-D) DiOC6 staining (mitochondria, green) and *dazl* in situ (E-H, red) in stage I oocytes label the Bb. (A, E) WT with centered nucleus and Bb present. (B, F) *buc* mutant with centered nucleus, absent Bb, and unlocalized *dazl*. (C, G) *macf1a* mutant with acentric nucleus and Bb enlarged. (D, H) *macf1a*^*p6cv*^*; buc* double mutant with acentric nucleus, absent Bb, and unlocalized *dazl*. DAPI (blue) stains DNA (A-D). DiOC_6_: N≥ 3 ovaries; >30 WT, >30 *macf1a*^*p6cv*^, 15 *buc*^*p106*re^, 24 *macf1a*^*p6cv*^*; buc*^*p106re*^ oocytes. *dazl in situ*: N = 3 ovaries; 10 *buc*^*p106re*^, 7 *macf1a*^*p6cv*^, 14 *macf1a*^*p6cv*^*; buc*^*p106re*^ oocytes. Representative images from 2 experiments. Dotted white lines outline the nucleus. Images are a sum of 3 single optical confocal sections. Arrowheads indicate the Bb. Scale bar: 20 μm.

Mutant *macf1a* oocytes display both an asymmetrically positioned nucleus and a Bb dissociation defect. These defects may represent two independent functions of Macf1a, one in Bb disassembly and one in nuclear positioning, or one of the defects may be a secondary effect caused by the other defect. For example, the enlarged, persistent Bb of *macf1a* mutants could displace the nucleus, causing it to become acentric. To determine if the Bb defect causes the nucleus to become asymmetric, we generated double mutants of *macf1a* and the *buc* mutant, which never forms a Bb. We hypothesized that if Macf1a functions independently in Bb disassembly and nuclear positioning, then a *buc* ^*p106re*^*; macf1a* ^*p6cv*^ double mutant should display an absence of the Bb and a nuclear positioning phenotype. However, if the Bb is absent and the nucleus is no longer acentric in the double mutant, then the nuclear defect can be considered a secondary effect of the Bb defect in *macf1a* mutants. We analyzed *buc; macf1a* double mutant oocytes and found that the Bb was absent, while the acentric nuclear phenotype remained (Fig 2A–2H). These results strongly support Macf1a functioning independently in Bb disassembly and nuclear positioning.

**Fig 3:**
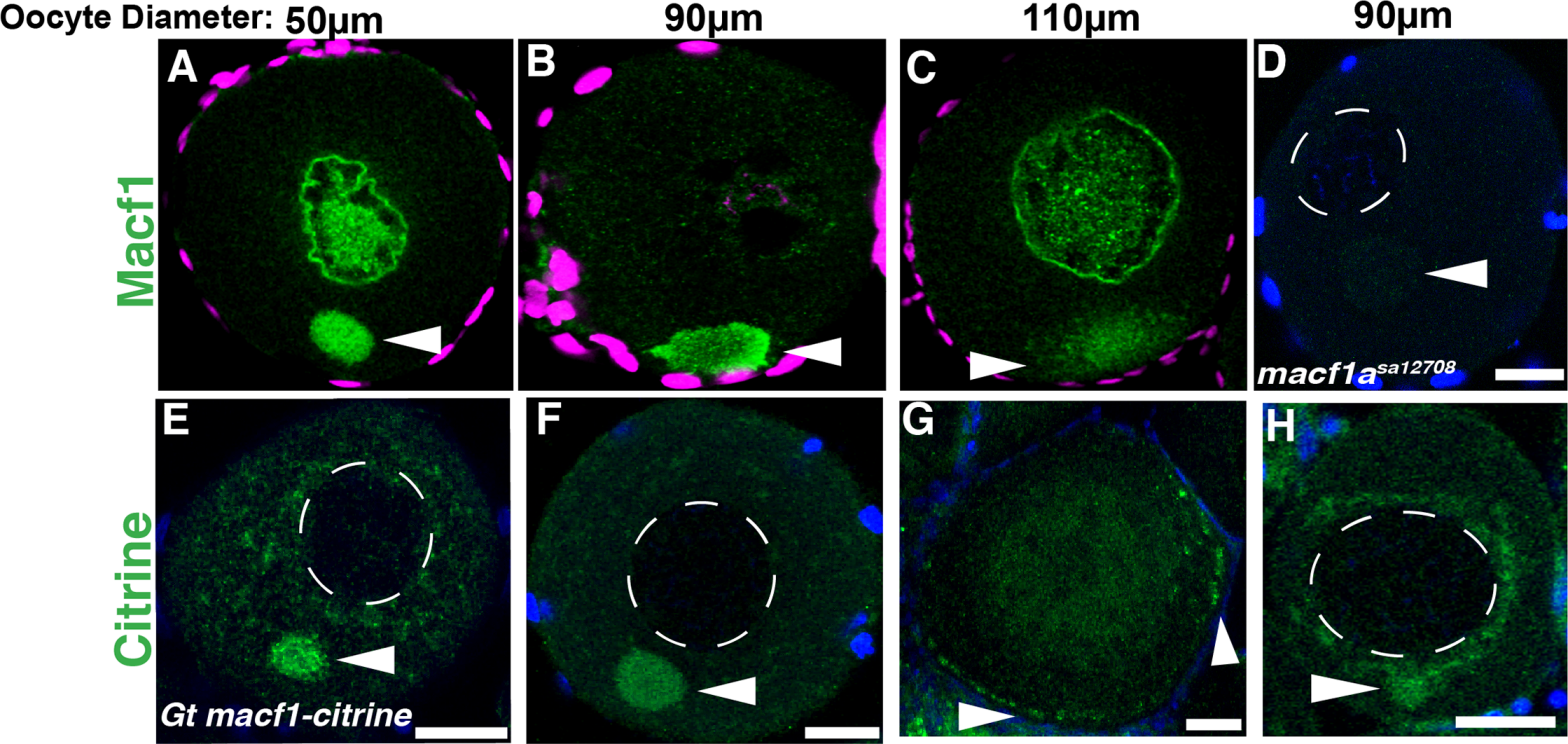
Localization of Macf1a in stage I oocytes. A-C) Macf1a immunostaining (green) in stage I oocytes shows localization nuclearly and within the Bb (A), and later at the cortex during Bb disassembly (B, C). D) Macf1a staining was negative in a *macf1a*^*sa12708*^ mutant oocyte. E-G) GFP staining in *Gt(macf1a–citrine)*^*ct68a*^ line also showed Macf1a-Citrine localization to the Bb and at the cortex during disassembly. DAPI (blue/magenta) stains DNA. A-D) N≥ 3 ovaries, 20 isolated WT oocytes were examined (see methods), 16 oocytes showed Macf1 nuclear staining; 18 *macf1a*^*sa12708*^ oocytes were examined. E-H) N = 3 ovaries, 9 of 20 *Gt(macf1a–citrine)*^*ct68a/+*^ oocytes showed nuclear staining (H). Representative images from 3 experiments. Dotted white lines outline the nucleus. Images are a sum of 3 single optical confocal sections. Arrowheads indicate the Bb. Scale bars: 20 μm.

We next tested if Macf1a regulates Bb disassembly and nucleus positioning directly by associating with the Bb and nucleus or if it acts via an indirect mechanism, for example, by localizing to the oocyte cortex and regulating these processes. To investigate this question, we examined the intracellular localization of Macf1a protein in stage I WT oocytes using an antibody against mouse Macf1 [9] and took advantage of a zebrafish gene trap line, Gt*(macf1a-citrine)*^*ct68a*^, inserted in a *macf1a* intron between exons 57 and 58 [29]. Using the Macf1 antibody, we found that Macf1a localizes to the Bb and nuclearly (Fig 3A). Importantly, no immunostaining was observed in the *macf1a*^*sa12708*^ mutant allele, indicating that the antibody is specific to Macf1a (Fig 3D). In later stage I oocytes, Macf1a localization recapitulated the dynamics of Bb disassembly at the vegetal cortex, progressively dissociating from the Bb and localizing to the oocyte cortex (Fig 3A–3C).

Immunostaining for the Citrine insertion in Macf1a also showed similar localization to the Bb and followed its dynamics (Fig 3E–3G). Perinuclear localization was also observed in about half the oocytes (9/20) (Fig 3H), though it did not fully recapitulate the nuclear distribution observed with the Macf1 antibody. We postulate that Macf1 levels are lower in the nucleus than in the Bb based on our ability to detect nuclear Macf1 only in conditions of high Macf1 antibody concentrations (see methods), whereas Macf1 in the Bb is consistently detected using high and lower Macf1 antibody concentrations (discussed further later related to testing the function of the Macf1-ABD). Importantly, these results support a model where Macf1a functions directly in the Bb to regulate its dissociation and anchoring to the oocyte cortex. Macf1a localization to the nucleus suggests a direct role for it in positioning the nucleus as well.

### Actin required for Bb cortical attachment and nuclear positioning, whereas MTs dispensable

**Fig 4:**
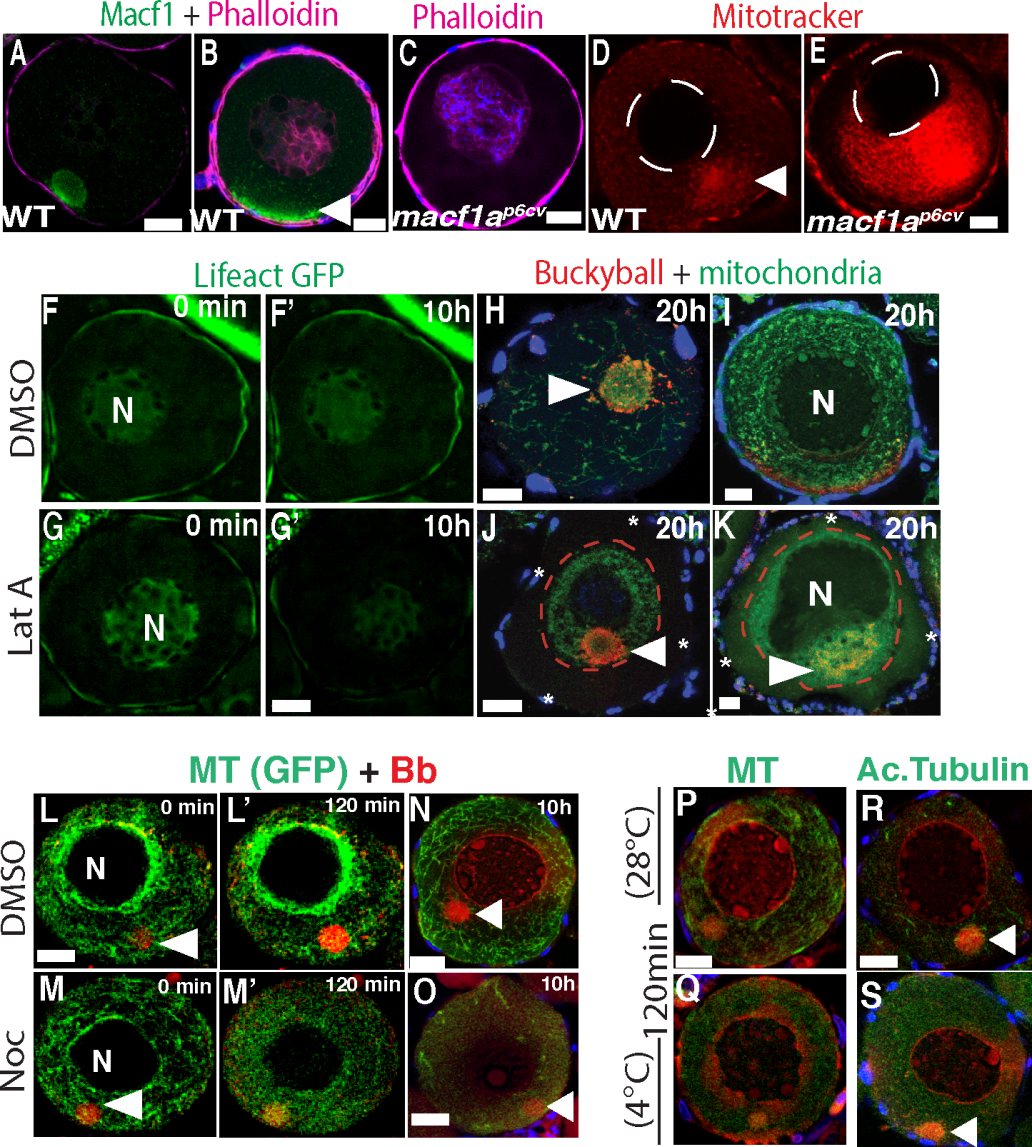
Effect of disrupting actin and MTs on the Bb and nuclear positioning. A-B) Macf1 (green) and phalloidin staining in WT, and C) phalloidin in *macf1a*^*p6cv*^ mutant oocytes labeling cortical and intranuclear actin. N = 3 ovaries, 5 WT and 10 *macf1a*^*p6cv*^ mutant oocytes. WT (D’) and *macf1a*^*p6cv*^ mutant (E) live stage I oocytes stained with MitoTracker to visualize the cortical detachment of mitochondria in *macf1a* mutant. N = 3 ovaries, 22 WT and 7 *macf1a*^*p6cv*^ oocytes. F-G) Live imaging of Lifeact-GFP ovaries treated with DMSO (F-F’) or LatA (G-G’) for 10h. G’) GFP signal decreases indicating acting disruption. H-K) Dissected ovaries enriched for stage 1 and 2 oocytes were treated with DMSO (H-I) or LatA (J-K) for 20 h, then fixed and stained for mitochondria (DiOC6, green) and Buc (red). Asterisks and red dotted line mark the cortical detachment of mitochondria and Buc in LatA treated oocytes (J-K). The number of oocytes imaged that showed the mitochondrial cortical detachment in DMSO and LatA treated was 3/25 and 25/40, respectively. F) The oocytes that displayed an acentric nucleus after LatA treatment was 15 of 40 oocytes. N ≥ 5 ovaries. L-M) live imaging of stage I oocytes Tg*(ef1a*:*dclk-GFP)* to visualize MTs (green) and Mitotracker (mitochondria, red) to visualize the Bb. Oocytes were treated with DMSO (L, control) or nocodazole (M). N≥5 ovaries, 75 DMSO and 73 nocodazole treated oocytes. N-O) Ovaries from Tg (EMTB-3GFP) treated with DMSO (N) or nocodazole (O) for 10h, and stained for MTs (GFP) and mitochondria (DiOC₆). N = 3 ovaries, 10 oocytes DMSO treated and 11 nocodazole treated. P-S) Ovaries incubated at 28°C (P,R) or 4°C (Q,S) to depolymerize stable MTs, were fixed after 120 min cold treatment and stained for MTs (P, Q; Tg*(ef1a*:*dclk-GFP))* or acetylated tubulin (R, S). N≥5 ovaries, 53 (28°C) and 50 (4°C) oocytes. Arrowheads indicate the Bb. Scale bar: 20 μm.

Macf1a contains binding domains for several different cytoskeletal elements and acts to integrate cytoskeletal systems in other models, thus we expect that Macf1a interacts with the oocyte cytoskeleton to regulate Bb dissociation and nuclear positioning. To determine which cytoskeletal components interact with Macf1a, we examined the distribution of actin and MTs in stage I oocytes. With this purpose, we performed live imaging experiments using the transgenes Tg*(actb1*:*lifeact-GFP)* [30] and Tg*(ef1a*:*dclk-GFP)* [31] to visualize actin and MTs, respectively. As shown in live and fixed samples, actin appeared as a thick cortical layer and intranuclearly, but was not present in the Bb in stage IB oocytes (Fig 4A, 4F and 4G) [5]. Actin localized similarly in *macf1*^*p6cv*^ mutant oocytes (Fig 4C). Upon Bb disassembly, Macf1 spreads at the cortex and is closely associated with cortical actin (Fig 4B).

We then tested the function of cytoskeletal components in stage I oocytes using pharmacological inhibitors. To test actin function, we disrupted actin filaments with Latrunculin A (LatA) and evaluated its effect on the Bb and nuclear positioning. After 6 hours (h) of treatment, we found that actin filaments were moderately affected (S1A and S1E Fig), and only after 10-12h of LatA treatment were actin filaments greatly reduced (Fig 4F–4G’, S1B and S1F Fig). We treated ovaries with LatA for 12h or 20h, then fixed and stained for Buc. After 12h of LatA treatment, Buc appeared detached from the oocyte cortex in a few oocytes (4/22), three of which also displayed an acentric nucleus (3/22 oocytes) (S1G and S1H Fig). After 20h of LatA treatment these effects were stronger. In control conditions Buc remained in the Bb or at the cortex (Fig 4H–4I), while in LatA treated oocytes the Bb was closer to the nucleus as in a pre-disassembly stage (Fig 4J–4K). Additionally, the nucleus was acentric in many oocytes (15/40) (Fig 4K). It is possible that the stronger effect of 20 versus 12h of LatA treatment is due to actin filaments that remain but are undetectable after 12h of treatment, which are sufficient to preserve Buc at the cortex, but are effectively disrupted after 20h of treatment. Thus, disruption of actin partially phenocopies the *macf1a* mutant phenotype (Fig 1B), suggesting that Macf1a may interact with cortical actin to mediate Bb disassembly and nuclear positioning.

In agreement with previous reports on fixed tissue in Xenopus [32], MT networks in live oocytes were present throughout the cytoplasm and were enriched perinuclearly (Fig 4L). Though we detected stable MTs (acetylated) in the Bb in some oocytes (Fig 4R, 10/25 oocytes), they were not enriched there. We addressed MT function by live imaging of ovaries treated with nocodazole for 2 and 10 h, or by incubating them at 4°C to also depolymerize stable MTs. In both cases, we found that depolymerization of MTs did not affect the Bb or nuclear positioning (Fig 4L–4S, S1–S4 Movies). This suggests that MTs do not play a role in regulating the Bb structure, nuclear positioning or in cortical attachment like we observed for actin.

**Fig 5:**
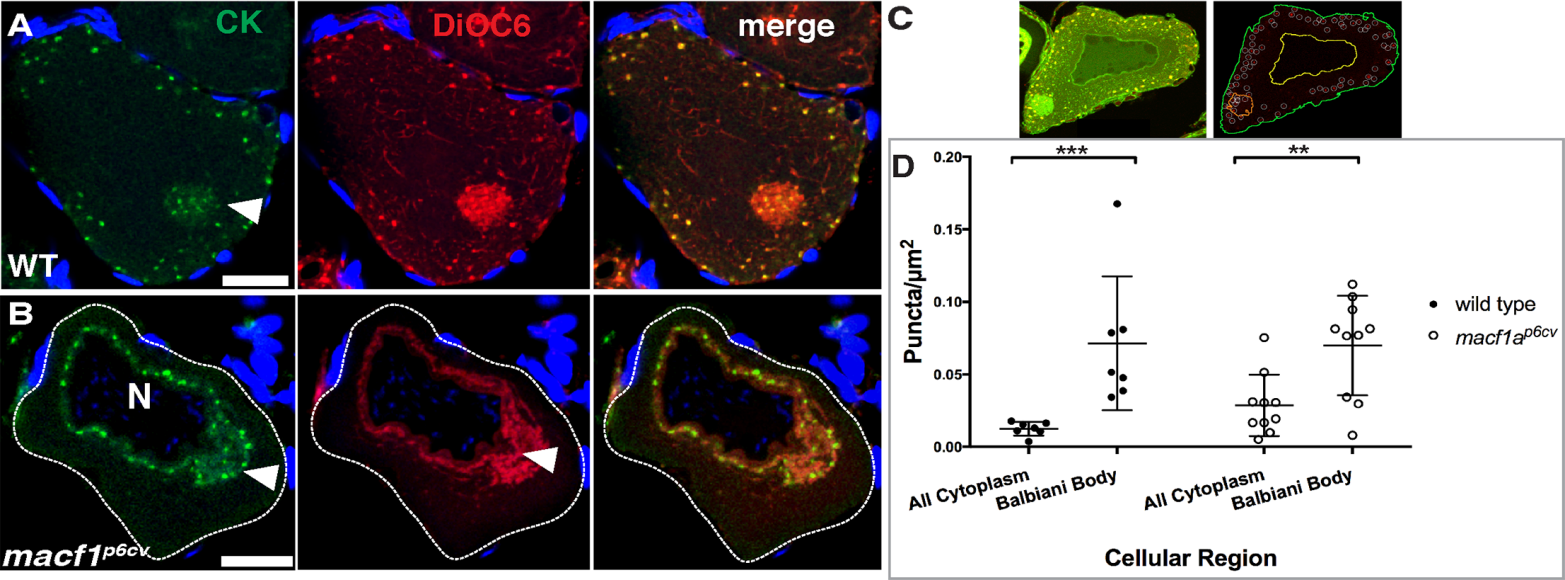
Distribution of cytokeratin in stage I oocytes. A-B) Cytokeratin (CK, green) immunostaining and DiOC6 labeling (Bb, red) in ovary tissue sections. A) In WT, CK puncta are distributed within the Bb (arrowhead) and cortically, whereas in *macf1a*^*p6cv*^ mutant oocyte (B-C) CKs are devoid from the cortex (white outline) and are around the nucleus. C-D) Quantification of CK enrichment in the Bb. C) DiOC6 staining pattern was used to segment the oocyte into regions of interest (ROI). Outlines show identified plasma membrane (green), nucleus (yellow) and Bb (orange). All cytoplasm defined by the area between yellow and green lines. White circles around identified CK puncta. CK puncta density was measured in each region of interest (ROI). Images are representative of 3 experiments. DAPI (blue) stains the follicle cell nuclei (A-B). Images are single optical sections. Scale bar: 20 μm. N ≥ 5 ovaries, > 30 WT and > 20 *macf1a*^*p6cv*^ oocytes. D) Graph of CK enrichment (CK puncta/μm^2^) in ROIs. N = 2 ovaries, 7 wild type and 10 *macf1a*^*p6cv*.^oocytes were used for quantification. Error bars, standard error of the mean. Black bar shows the mean. P values are calculated for ROI differences by two-way ANOVA and Tukey's Multiple Comparison Text to compare between genotypes. ** P < 0.01; *** P < 0.001.

Finally, we examined the distribution of IF using a Pan-Cytokeratin (CK) type II antibody. We found that CK was distributed in a punctate pattern in the Bb and cortically in stage 1B WT oocytes (Fig 5A). We quantified the CK distribution in the Bb in WT using a custom MATLAB program and found that CK puncta density is significantly enriched in the Bb (4-fold increase) compared to the cytoplasm (Fig 5C and 5D). Interestingly, in *macf1a* mutants CK puncta were still significantly enriched in the Bb, though cytoplasmic CK appears accumulated around the nucleus, similar to the detachment of other components from the cortex in *macf1a* mutants (Fig 5B and 5D). Thus, Macf1a may function in Bb disassembly via integrating CK in the Bb to actin at the cortex, and CK localization to the Bb is likely Macf1a independent.

### Genome editing approach to interrogate Macf1a functional domains

The modular domain structure of Macf1a allows us to interrogate Macf1a by targeting specific cytoskeleton-binding functions. We postulated that Macf1a mediates Bb disassembly by binding cortical actin via its Actin binding domains (ABDs) and by binding CK within the Bb via its Plectin repeat domain (PRD), integrating the cortical actin and CK and therefore disassembling the Bb at the oocyte cortex. Results supporting this hypothesis are that CK and Macf1a are both enriched in the Bb and actin depolymerization disrupts Bb cortical anchoring. In addition, because we observed Macf1a localized to the nucleus and disruption of actin causes acentric nuclear positioning, the Macf1a-ABDs may also be required to position the nucleus in the oocyte.

**Fig 6:**
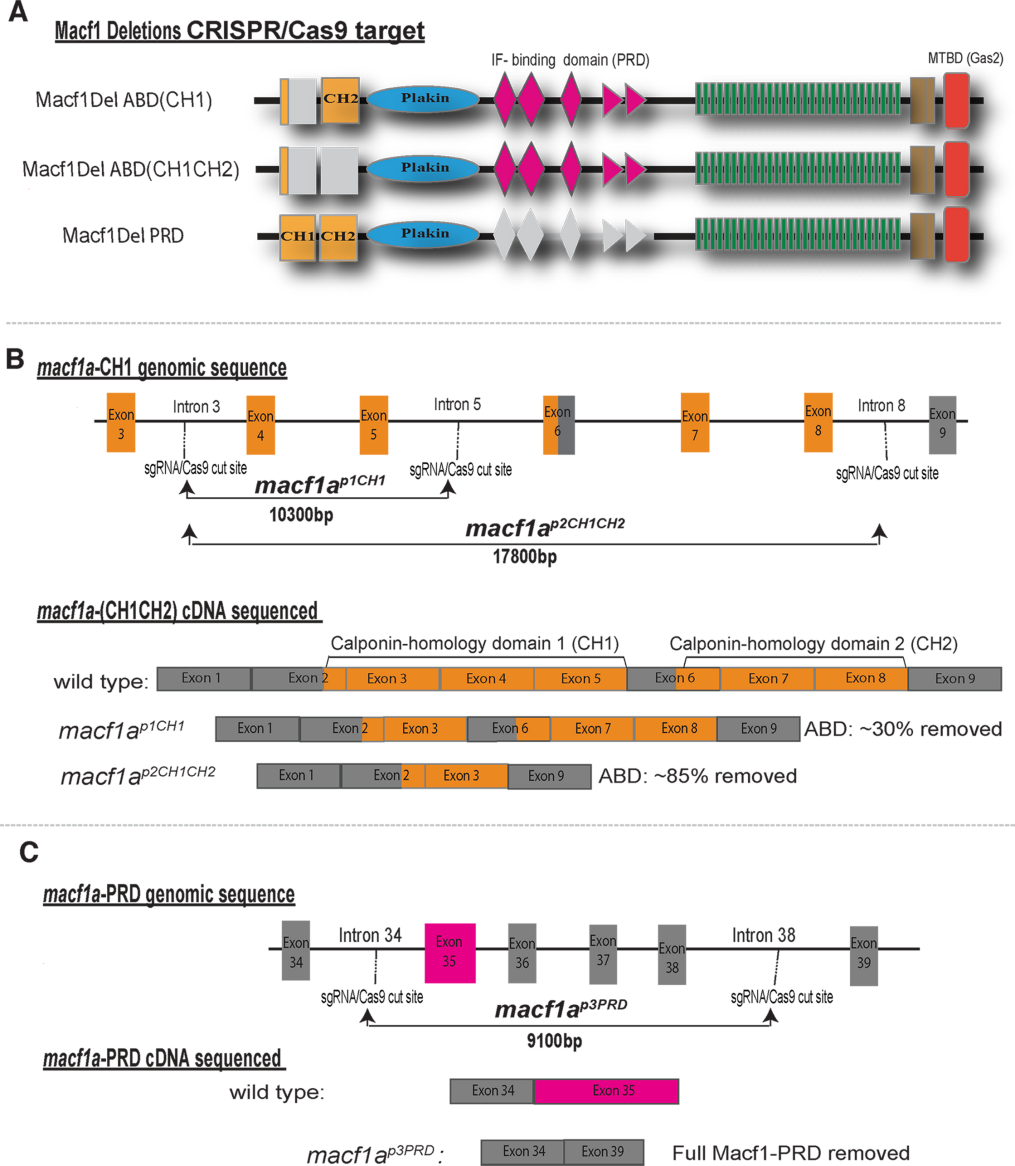
CRISPR/Cas9 deletions of Macf1a ABD and PRD. A) Illustrates the Macf1a protein generated by the genomic deletions and the nomenclature to refer to each deletion mutant. Exons encoding the ABD are highlighted in yellow and the PRD in pink, whereas the absent domains are in gray. B-C) Schematizes the CRISPR/Cas9 approach to delete the ABD and PRD, showing the targeted introns, the size of the genomic deletion, and the deleted exons from the coding sequence (cDNA). B) Sequencing of cDNA confirmed deletion of intended exons coding for 70% of the CH1 ABD and 85% of the full CH1-CH2 (ABDs). C) Illustrates the deletion of exon 35, coding for the PRD, and exons 36–38.

To interrogate Macf1a-ABD and -PRD functions in oocyte polarity, we developed a CRISPR/Cas9 approach to make large deletions in the endogenous *macf1a* gene. We designed sgRNAs targeting the introns flanking the ABD and the PRD encoding exons to remove each domain (Fig 6, and see methods). Importantly, the deleted exons did not alter the reading frame of the *macf1a* ORF. Our strategy was to first inject each sgRNA singly with Cas9 protein into 1-cell stage F0 embryos to confirm high frequency cutting (Table 1). Cutting efficiency was assayed by PCR amplification of genomic DNA spanning the target site, followed by high resolution melt analysis (HRMA) of the PCR product [33]. We increased the amount of sgRNA injected until every F0 embryo showed CRISPR-induced HRMA shifts. Then we targeted specific *macf1a* domains by simultaneously injecting two sgRNAs with Cas9 into 1-cell stage embryos. We optimized the sgRNA concentration further o obtain high frequencies of deletions without embryo abnormalities. We detected the deletions by PCR analysis of genomic DNA, then sequenced the PCR products to confirm that the deletions were consistent with our predictions. We raised F0 injected fish and tested for germline transmission in F1 embryos. Since F0 fish display germline mosaicism for the induced mutations, we do not expect Mendelian ratios in the F1 (Table 1). All future generations examined displayed normal Mendelian ratios of homozygotes, heterozygotes and wild-types.

**Table 1:** Generation of *macf1a* deletion mutants.

Targeted *macf1a* introns (I) and domains	sgRNA injected	^1^Indel efficiency	Deletion size	^2^Positive F0/Total tested	^3^Positive F1 embryos/Total tested	Positive adult F1/Total F1
I3+I5 Calponin-homology 1 (Actin-binding)	I3: 550 pg I5: 325 pg	I3: 96% I5: 87.5%	10.3 kb	1/9	3/24	3/30
I3+I8 Calponin-homology 1&2 (Actin-binding)	I3: 550 pg I8: 466 pg	I3: 96% I8: 83%	17.8 kb	1/28	1/4	3/20
I34+I38 PRD (IF-binding)	I34: 415 pg I38: 400 pg	I34: 82.5% I38: 90%	9.1 kb	1/15	6/18	4/24

^1^ The percent corresponds to F0 embryos that showed indels from the total number of injected embryos examined.

^2^ Ratio represents the number of F0 adult females that showed germline transmission in their F1 for the indicated deletions.

^3^ The number of F1 embryos carrying the deletion from the total F1 embryo progeny tested.

The Macf1a-ABD is composed of two calponin-homology (CH) domains: CH1 and CH2. A single CH domain can bind actin, but many actin binding proteins contain two CH domains. Macf1 and some Plakin isoforms in other species contain either the full CH1-CH2 ABD or the CH2 domain alone [34]. We designed CRISPR sgRNAs to generate deletions that would recapitulate the known Spectraplakin isoform lacking CH1, as well as to test if the full ABD functions in Bb disassociation and nucleus positioning. Thus, we targeted the CH1 domain, as well as both CH1and CH2 domains. To delete *macf1a*-CH1 we targeted introns 3 and 5, deleting exons 4 and 5, which spans 70% of the *macf1a*-CH1 coding sequence (Fig 6A and 6B). To delete *macf1a*-CH1-CH2 we targeted introns 3 and 8, removing exons 4–8, which encompass 85% of the entire *macf1a*-CH1-CH2 (Fig 6B). We succeeded in deleting 10.3 kb and 17.8 kb of genomic DNA to remove the Macf1a-CH1 and the Macf1a-CH1-CH2, respectively, through germ line transmitted mutations (Fig 6A and 6B, S2A and S2C Fig).

The Macf1a-PRD is entirely contained within exon 35 (NCBI: XP_001920094.1). We targeted the immediately preceding intron 34; however, due to the small size of introns 35–37, and to preserve the transcript ORF, we chose intron 38 as the second CRISPR target for deleting the PRD (Fig 6A and 6C). Importantly, the 174 amino acid region encoded by exons 36–38 that were targeted for deletion does not appear to include key components of Macf1a functional domains (based on a SMART domain prediction). We succeeded in deleting 9.1 kb of genomic DNA to remove the Macf1a-PRD in a germ line transmitted allele (Fig 6C, S2B and S2C Fig).

From the *macf1a* ^*p1CH1*^ and *macf1a* ^*p2CH1CH2*^ mutants, we sequenced ovary cDNA spanning exons 3 to 8, which encode the ABD, and confirmed the lack of the intended exons in *macf1*^*p1CH1*^ and *macf1a* ^*p2CH1CH2*^ (Fig 6B, S2A Fig). For the PRD, we detected in both WT and *macf1a*^*p3PRD*^ mutant oocytes a small RT-PCR band (~750 bp) (S2C Fig) that sequence analysis showed corresponds to an alternatively spliced transcript that does not include exons 35–39, as discussed above (*macf1a* cDNA results). In addition, we detected a larger band (970bp) only present in the *macf1a*^*p3PRD*^ mutant that sequence analysis showed is generated by the deletion of exons 35 (PRD) to 38. We amplified in ovary cDNA an ~1.5 kb band from the flanking exon 34 into exon 35 in WT (confirmed by sequence analysis), which was absent in *macf1a*^*p3PRD*^ mutants (S2C Fig). Together, these results confirmed that we deleted the PRD from the *macf1a* endogenous locus and, generated a transcript lacking the Macf1a-PRD.

In summary, we deleted the exons encoding the Macf1a ABD and PRD from the *macf1a* gene using CRISPR/Cas9 technology. The transcript produced from the genome-edited mutants lack these specific domains but preserve the *macf1a* transcript ORF.

### Macf1a-ABD is essential for Bb disassembly and nuclear positioning

**Fig 7:**
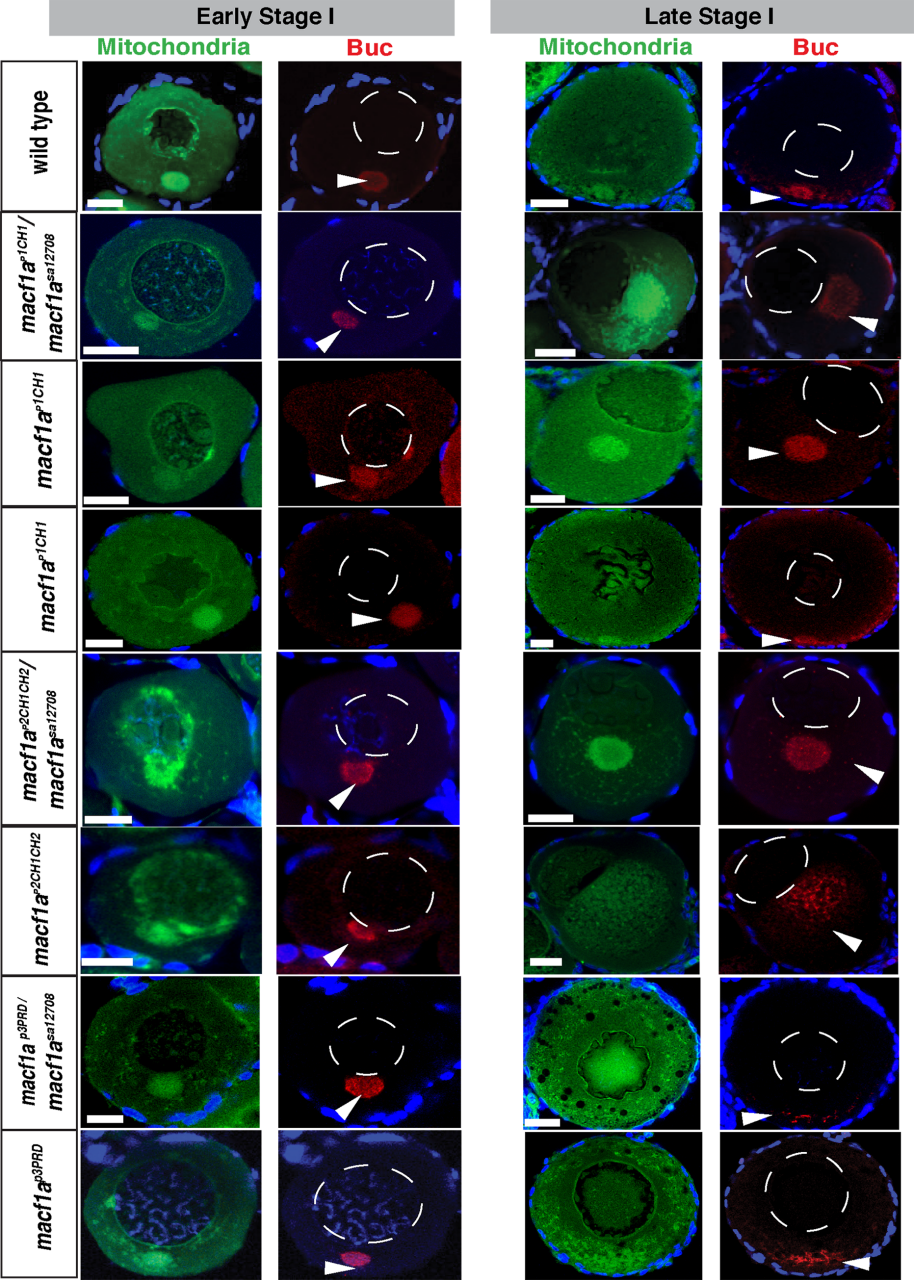
Characterization of *macf1a*^*p1CH1*^, *macf1a*^*p2CH1CH2*^ *and macf1a*^*p3PRD*^ *mutants*. DiOC_6_ (mitochondria, green) and Buc (red) staining in early and late stage I oocytes. *macf1a*^*p1CH1*^ */macf1a*^*sa12708*^ and *macf1a*^*p2CH1CH2*^ /*macf1*^*sa12708*^ ovaries display a *macf1a* null phenotype, whereas *macf1a*^*p*3*PRD*^ */ macf1*^*sa12708*^ show no phenotype. *macf1a*^*p1CH1*^ ovaries showed incomplete penetrance, displaying either a WT or a *macf1a* null phenotype. *macf1a*^*p2CH1CH2*^ oocytes displayed a *macf1a* null phenotype, and *macf1a*^*p*3*PRD*^ showed no phenotype. DiOC_6_ and Buc; N≥ 3 ovaries of each genotype, 31 *macf1a*^*p1CH1*^, 25 *macf1a*^*p1CH1*^***/****macf1a*^*sa12708*^, 11 *macf1a*^*p2CH1CH2*^***/*** *macf1a*^*sa12708*^, 22 *macf1a*^*p2CH1CH2*^, 15 *macf1a*^*p*3*PRD*^*/macf1*^*sa12708*^ and 24 *macf1a*^*p*3*PRD*^ oocytes. DAPI (blue) stains the DNA. Dotted white lines outline the nucleus. Images are single optical sections. Arrowheads in indicate the Bb, and in B the egg cytoplasm. Scale bar: 20 μm.

**Fig 8:**
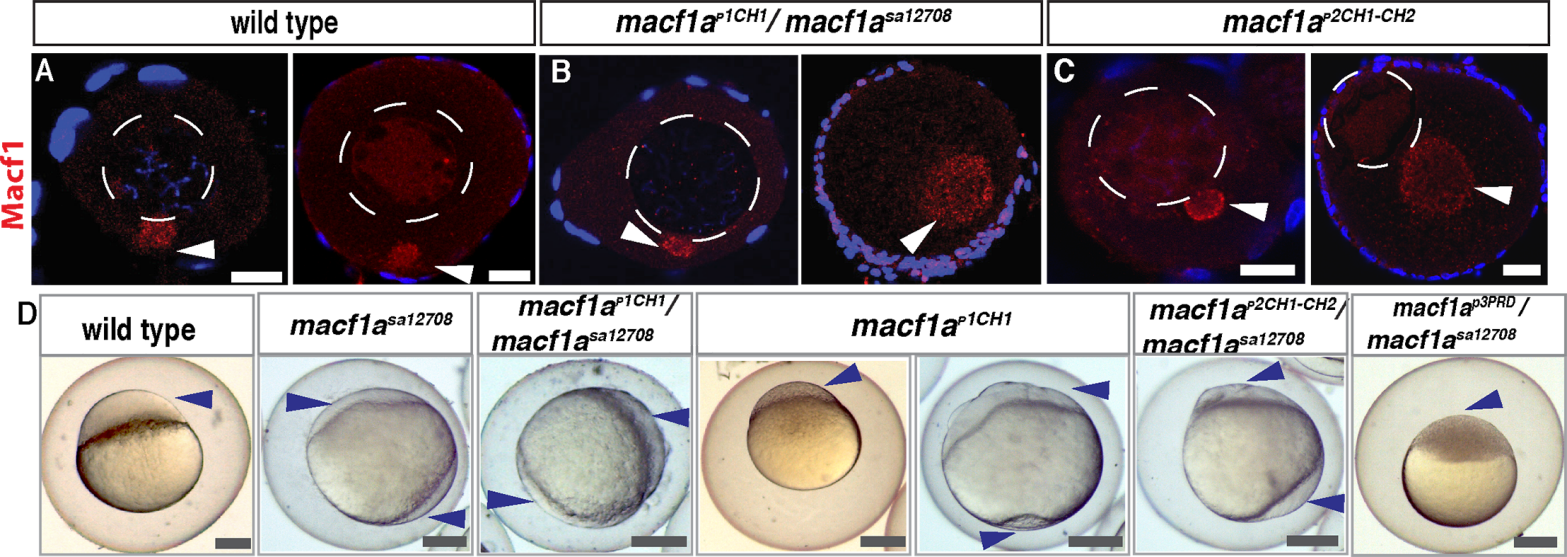
Macf1 localization and expression in *macf1a*^*p1CH1*^ *and macf1a*^*p2CH1CH2*^ *mutants*. A-C) Macf1a immunostaining (red) in WT and *macf1a*^*p1CH1*^*/macf1a*^*sa12708*^ *and macf1a*^*p2CH1CH2*^. N≥ 3 ovaries, 20 oocytes *macf1a*^*p1CH1*^*/macf1a*^*sa12708*^ and 28 oocytes *macf1a*^*p2CH1CH2*^. D) AV polarity in WT and lack of polarity in *macf1a*^*sa12708*^, *macf1a*^*p1CH1*^*/macf1a*^*sa12708*^ and *macf1a*^*p2CH1CH2*^/*macf1*^*sa12708*^ mutant eggs, where the cytoplasm (arrowheads) surrounds the yolk instead of forming the blastodisc. Mutant *macf1a*^*p1CH1*^ females produced an incompletely penetrant egg phenotype. Late blastula embryos from *macf1a*^*p*3*PRD*^*/ macf1*^*sa12708*^ and *macf1a*^*p*3*PRD*^ mutant females displayed normal AV polarity and development. Arrowheads in D indicate the egg cytoplasm. Scale bar: (A-C) 20 μm and (D) 100 μm.

To determine the role of the Macf1a ABD in Bb progression and nuclear positioning, we analyzed the ovaries of *macf1a* ^*p1CH1*^ and *macf1a* ^*p2CH1CH2*^ deletion mutants. First, we generated transheterozygotes of *macf1a* ^*p1CH1*^/*macf1a*^*sa12708*^ and *macf1a* ^*p2CH1CH2*^ */macf1a*^*sa12708*^ (see methods). We found that in both cases the oocytes displayed a fully penetrant *macf1a* null mutant phenotype: the nucleus was acentric and the Bb failed to disassemble in late stage I oocytes (Fig 7A). Then we examined homozygous mutant ovaries of each allele. We found that *macf1a* ^*p2CH1CH2*^ homozygous mutant oocytes showed a *macf1a* null phenotype. However, *macf1a* ^*p1CH1*^ homozygous mutant females exhibited an incompletely penetrant *macf1a* null phenotype, with ovaries from one female showing either a WT or mutant phenotype in the full set of oocytes (Fig 7A). Importantly, the Macf1a mutant protein was produced and localized to the Bb in both *macf1a* ^*p1CH1*^ and *macf1a* ^*p2CH1CH2*^ mutant oocytes as in WT, and it showed similar expression levels in immunostaining (Fig 8A–8C). We did not clearly detect Macf1 in the nucleus, most likely as a result of using lower Macf1 antibody concentrations for these experiments (see methods and discussion).

**Fig 9:**
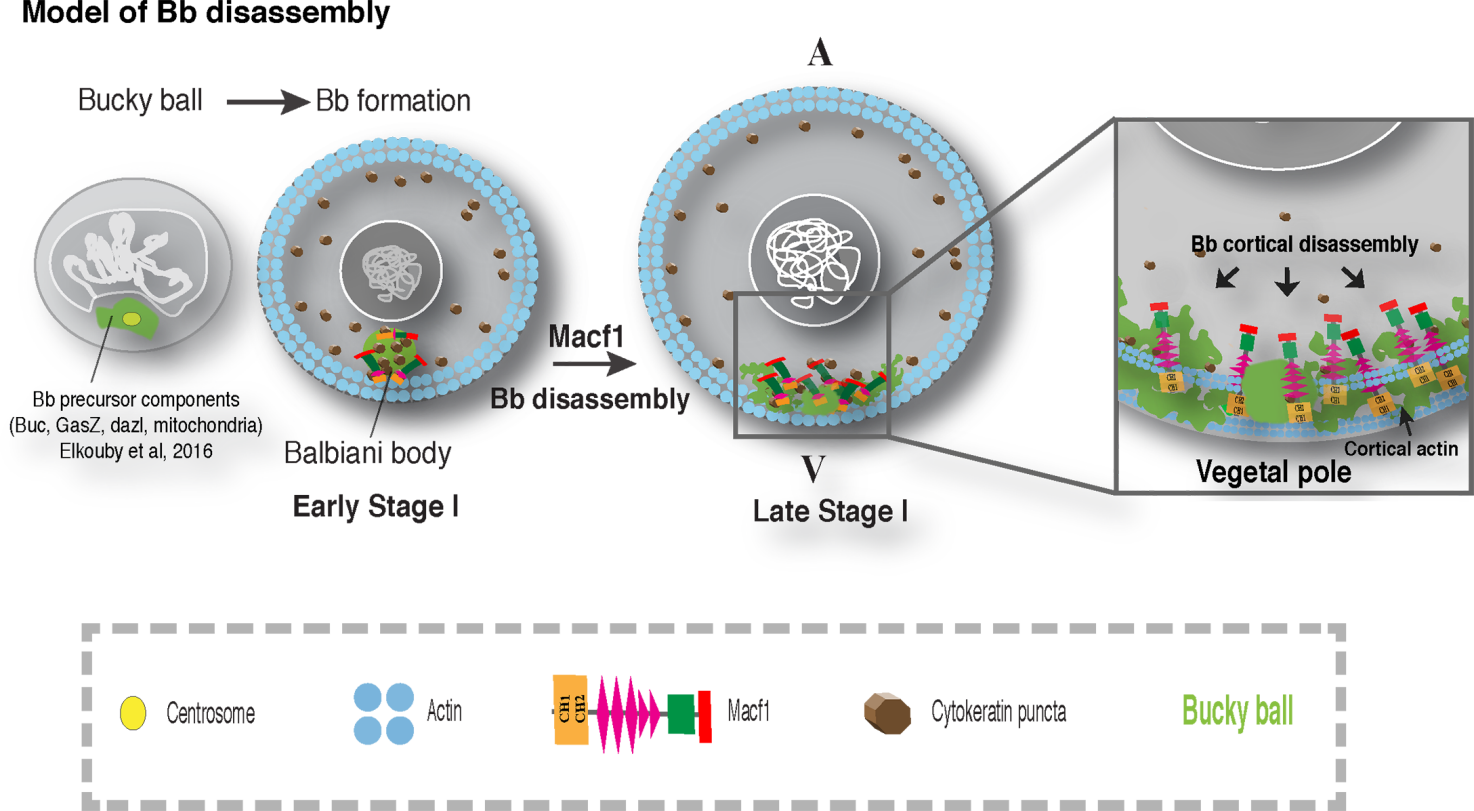
Model of Macf1a function in Bb dissociation at the cortex. In early stage I oocytes, Bb precursors first aggregate in a nuclear cleft around the centrosome. As stage I progresses the centrosome is lost and the Bb rounds up into a mature Bb (Elkouby et al 2016). From this position next to the nucleus the Bb will later reach the oocyte cortex. The mature Bb contains Buc and Macf1a that regulate Bb formation and disassembly, respectively. We postulate that Macf1a in the Bb does not function as a cytolinker linking two cytoskeletal systems together. Instead, Macf1a links the Bb to the oocyte cortex via its localization to the Bb and by interaction with cortical actin via the Macf1a ABD. The Bb dissociates progressively during a period of about two-fold growth in oocyte volume. During this period the Bb becomes progressively smaller, although remains largely spherical, with Macf1a, Buc and other Bb components relocating as puncta to the oocyte cortex. We hypothesize that Macf1a acts directly in this relocalization process, which may occur via dissolution and reaggregation or via the Bb fragmenting and relocalizing to the oocyte cortex in a Macf1a-dependent manner. In either case, the Bb would dissociate progressively over time with peripheral regions dissociating prior to more internal ones. Once the Bb dissociates at the cortex, the oocyte vegetal pole is defined.

Similar to the *macf1a*^*sa12708*^ mutant phenotype, Buc remains localized in the persisting, enlarged Bb of *macf1a* ^*p1CH1*^*/macf1a*^*sa12708*^, *macf1a* ^*p1CH1*^, *macf1a* ^*p2CH1CH2*^ /*macf1a*^*sa12708*^ and *macf1a* ^*p2CH1CH2*^ oocytes (Fig 7). AV polarity is also affected in the eggs of these mutant females (Fig 8D, Table 2). The incomplete penetrance of the *macf1a* ^*p1CH1*^ ovary mutant phenotype is also observed in the AV egg phenotype (Fig 7B, Table 2). These findings show that the Macf1a-ABD mediates Bb granule dissociation at the cortex, nuclear positioning, and is essential for defining the AV axis (Fig 9 model). Furthermore, both the CH1 and CH2 ABDs provide the robust function needed for Bb disassembly and nuclear positioning, whereas the CH2 domain alone can suffice but often is insufficient.

**Table 2:** Egg phenotype of *macf1a* mutant females.

Genotype	WT AV polarity, N° eggs*	No AV polarity, N° eggs	Total N° females
*Wild type*	46	0	2
*macf1a*^*sa12708*^	0	81	2
*macf1a*^*p1CH1*^/*macf1a*^*sa12708*^	4 (2.8)	142 (97.2)	4
*macf1a*^*p1CH1*^	319 (70)	137 (30)	9
*macf1a*^*p2CH1CH2*^*/macf1a*^*sa12708*^	5 (3.0)	160 (97.5)	4
*macf1a*^*p*3*PRD*^*/macf1a*^*sa12708*^	116	0	2
*macf1a*^*p*3*PRD*^	200	0	5

* In parenthesis is the percentage from total number of eggs

Unexpectedly, when we analyzed the ovaries of the *macf1a*^*p3PRD*^/*macf1a*^*sa12708*^ and *macf1a*^*p3PRD*^ deletion mutants, which lack the domain that can interact with IF, Bb disassembly was not affected (Fig 7A). In *macf1a*^*p3PRD*^ mutant oocytes, Buc localizes to the Bb normally and the nucleus is centrally located as in WT. Furthermore, eggs from *macf1a*^*p3PRD*^/*macf1a*^*sa12708*^ and *macf1a*^*p3PRD*^ homozygous mutant females display normal AV polarity and development (Fig 8D, Table 2). These results show that the Macf1a-PRD is not required to localize Macf1a to the Bb or to disassemble the Bb RNP granule. Thus the Macf1a-PRD is dispensable for Macf1a function in AV polarity and nuclear positioning.

## Discussion

### Macf1a may function as a non-canonical linker

Macf1a is a cytolinker that integrates cytoskeleton components in different cellular contexts [9, 10, 13, 20, 35]; however, a function for Macf1a in dissociating a large granule like the Bb is unprecedented, and so it is unclear how it may integrate MTs, actin, or IFs in Bb disassembly. Here, we developed a novel approach to unravel Macf1a domain-dependent function by interrogating, for the first time, the very large endogenous *macf1a* gene through CRISPR-Cas9 mediated exon deletions (Fig 6). We found that the Macf1a ABD is essential for Bb disassembly, whereas the IF interacting domain of Macf1a, the PRD, is dispensable. Since we did not detect MT enrichment in the Bb, nor a defect when MTs were depolymerized (Fig 4L–4S), it is possible that Macf1a does not function as a cytoskeletal linker in this context. Rather it may link Bb components to the oocyte cortex through its ability to bind cortical actin and localize to the Bb (Fig 9). Thus, it may function to link two structures together, but not through its canonical cytoskeletal specific cross-linking function.

### Macf1a in Bb disassembly, IDPs, and P granules

C. elegans P-granules have become an excellent model for studying the assembly/disassembly dynamics of RNP granules. P granules form via the assembly of MEG proteins, which are intrinsically disordered proteins (IDPs) acting like a scaffold for P granule condensation [36]. MEG is the target of kinases and phosphatases that regulate the disassembly and assembly of P granules, respectively, and are crucial in establishing the anterior-posterior axis of the embryo [36, 37]. Similarly, Buc and its ortholog in Xenopus Xvelo are IDPs functioning in Bb granule formation. The N-terminal region of Xvelo contains a prion-like domain required for Xvelo assembly in an amyloid-like matrix that can enclose Bb components [1]. In zebrafish oocytes, Bb cortical disassembly defines the future oocyte and egg vegetal pole, and Macf1a is the only known factor to function in this process. Our data show that Buc remains localized in a persistent Bb in *macf1a* mutants, suggesting that Macf1a either directly triggers Bb disassembly at the cortex or is required to mediate Bb component interaction with cortical factors that trigger Bb disassembly.

Our results show that deletion of the CH1-CH2 actin binding domains in an otherwise intact Macf1a protein that localizes to the Bb causes a failure in Bb granule dissociation. These defects are indistinguishable from those of the two *macf1a* nonsense alleles. A *macf1a* mutant lacking the CH1 binding domain, leaving intact the CH2 domain in *macf1a* ^*p1CH1*^/*macf1a*^*sa12708*^ transheterozygotes also exhibits a fully penetrant mutant phenotype; however, the phenotype shows incomplete penetrance in *macf1a* ^*p1CH1*^ homozygous mutants. This suggests that *macf1a* ^*p1CH1*^ is an hypomorphic allele, unable to restore function over the null allele (transheterozygous) but partially restoring function in a homozygous condition. Interestingly, Macf1 and other Spectraplakins generate isoforms lacking the CH1 ABD that bind actin with lower affinity [9, 38]. Although differences in genetic background may be affecting the penetrance of the phenotype, these results are consistent with the Macf1a-CH2 ABD having a lower affinity to actin that is frequently insufficient to link the Bb to cortical actin, revealing the functional importance of the conserved CH1-CH2 domains in Spectraplakins.

These results indicate that the Macf1a-ABD interacts with cortical actin to anchor the Bb to the oocyte cortex where it undergoes dissociation. Consistent with this, actin disruption phenocopied aspects of the *macf1a* mutant phenotype, causing the detachment of the Bb from the cortex. It is possible that Macf1a interaction with actin is sufficient to trigger Bb disassembly, or that other factors are required in addition to Macf1a. P granules, for instance, disassemble upon phosphorylation of structural components [36]; thus, Buc may be phosphorylated at the cortex in a Macf1a-ABD dependent manner.

Our analysis shows that the Bb does not disassemble all at once, but instead dissociates progressively during a period of about two-fold growth in oocyte volume. During this period the Bb becomes progressively smaller, although remains largely spherical, with Macf1a, Buc and other Bb components relocating as puncta to the oocyte cortex. We hypothesize that the Bb dissociates progressively with peripheral regions dissociating prior to more internal regions. During this process, it remains unclear whether Bb components fully dissolve at the cortex upon disassembly and then reassemble in aggregates that are docked cortically in a two- step process, or if the Bb fragments into smaller granules, with each granule anchored in a single step to the oocyte cortex. Either way, the Macf1a-ABD is a key activity in the process, which could be accompanied with modifications to Buc and/or other Bb components that act in Bb dissociation at the cortex.

### Drosophila Macf1 ortholog Shot and MTs

Shot, the Macf1 ortholog in Drosophila, also plays a key role in establishing oocyte polarity. Shot is required for anchoring MTs to the anterior and lateral oocyte cortex by interacting with the MT minus-end binding protein Patronin, which together with Shot functions as a noncentrosomal MT organizing center [39]. The Shot-ABD is required for its localization to cortical actin. The MT network organized by Shot/Patronin is key to setting up anterior-posterior polarity of the Drosophila oocyte. Both Macf1a and Shot function through their ABDs and localize with actin at the oocyte cortex, however, the mechanisms by which they act are likely distinct. In Drosophila, the assembly of MTs is downstream of Shot polarized cortical localization, which is regulated by Par-1, whereas Macf1a localizes at the oocyte cortex after Bb disassembly. Nevertheless, a role for Macf1a more broadly in linking MTs to the cortex is consistent with an absence of MTs at the cortex in zebrafish *macf1a* mutant oocytes [5], where it could act with Patronin to organize the MT network at a stage when the centrosome is absent [40].

Our live imaging data suggest that disruption of MTs does not affect the Bb. In keratinocytes and fibroblasts Macf1a localizes to MTs, and can function as a plus tip binding protein, stabilizing them [9, 10]. Also in zebrafish hair cells, the expression of the Macf1a-Citrine fusion in *Gt(macf1a–citrine)*^*ct68a*^ line shows that Macf1a colocalizes with actin and microtubules [14]. However, MTs are not enriched in the Bb and disruption of the MT network with nocodazole and cold treatment does not affect Bb morphology, RNA localization or cortical attachment (Fig 4L–4S) [8, 25, 32, 41, 42]. Thus, we do not consider a role for MTs or the Macf1a-MTBD currently in Bb disassembly. Nevertheless, since Macf1a stabilizes MTs and connects them to cortical actin in other cell types [9, 10, 13], we cannot rule out a contribution of MTs, until the conditions for culturing zebrafish oocytes allow in vivo visualization of Bb disassembly dynamics and the role of MTs in the process can be addressed. Future studies interrogating the Macf1a-MTBD will also clarify its contribution in oogenesis.

### Macf1a in nuclear positioning

We found here that Macf1a localizes to the nucleus in the oocyte, suggesting that Macf1a plays a direct role in positioning the nucleus centrally in the oocyte. We postulate that Macf1a nuclear concentration is lower than in the Bb, as evidenced by the absence of Macf1a nuclear staining in conditions of lower Macf1 antibody concentration (Fig 8 versus Fig 3, see methods). Our observation is supported by a recent study describing the nuclear proteome of frog oocytes, that quantified the nucleocytoplasmic partitioning of ~9,000 proteins [43]. Among those is Macf1, showing a nucleocytoplasmic partition of ~0.08, suggesting that Macf1 is ~12X higher in the cytoplasm than in the nucleus. Further evidence from the *macf1a; buc* double mutant shows that the nuclear localization defect is not caused by the Bb defect (Fig 2). Macf1a may interact with proteins residing in the nuclear envelope (NE) to connect the nucleus to the cytoskeleton. Although Nesprins residing in the outer NE interact with MTs to regulate nuclear positioning [44–50], our data indicate that disruption of MTs does not affect nuclear positioning. Rather, we found that actin disruption often leads to acentric nuclear positioning (Fig 4K), albeit we did not detect actin filaments around the nucleus and instead as an intranuclear mesh. Considering that the Macf1a ABD is required for nuclear positioning, it is possible that it interacts with cytoplasmic or perinuclear actin filaments that are undetectable in our conditions or, alternatively, that the Macf1a ABD interacts with a yet unknown partner in positioning the nucleus. Future studies will be required to deduce the mechanism.

In summary, we developed a genome editing approach using CRISPR-Cas9 to interrogate the cytolinker function of Macf1a. With this approach, we identified the Macf1a ABD as essential for Bb RNP granule disassembly and, thus, for establishing AV polarity of the oocyte and egg. The Macf1a ABD is also required for centric nucleus positioning in the oocyte. Moreover, we determined that the Macf1a PRD is dispensable for Macf1a function in oogenesis indicating that Macf1a does not interact with CKs to regulate Bb dissociation or nucleus localization, though we cannot rule that other Macf1 domains may be interacting with CKs. This is the first study to address Macf1a functional domains by targeting its endogenous locus. Although the use of CRISPR technology is widespread nowadays, applications like the one presented here take a step further in developing more powerful and elegant genome editing approaches than solely generating null alleles, to reveal a deeper understanding of basic cellular mechanisms. We expect that applying similar strategies will be valuable to understand the function of other modular proteins like Macf1, including the related genes *dystonin* [51], *dystrophin* [52] and *plectin* [53], which like *macf1* [54] can lead to complex human diseases.

## Materials and methods

### Ethics statement

All animal studies were approved by the University of Pennsylvania Institutional Animal Care and Use Committee (Protocol number 804214). Animal care and use adhered to the National Institutes of Health Guide for the Care and Use of Laboratory Animals.

### Fish lines

Ovaries were collected from 3 to12 month old adult fish of TU wild type, *macf1a*^*p6cv*^ [5], *macf1a*^*sa12708*^ (Sanger Center Mutation Resource), *buc*^*p106re*^ [2, 3], Tg*(ef1a*:*dclk-GFP)* [31]*, Tg(bactin2:HsENSCONSIN17- 282-3xEGFP) [55], Tg(actb1:lifeact-GFP)* [30] and *Gt(macf1a–citrine)*^*ct68a*^ [29]. For genotyping we used the following primers and PCR conditions:

*macf1a*^*p6cv*^ primers, Forward (For): GCCGACGACCACTTTTAGAG, Reverse (Rev): CCTGTCTGCCATCCTCAAAC. Denaturing: 94°C, 1:00 min. Annealing: 58°C, 45 seconds (sec) Extension: 72°C, 45 sec X 30 cycles. PCR product wild type: 201bp, *macf1a*^*p6cv*^: 170bp. Run in 3% agarose gel (50% of the agarose is Metaphor agarose).

For *macf1a*^*sa12708*^ genotyping, we used the KASPar^TM^ genotyping protocol of LGC Genomics [56]. KASPar sequence: CTGGTAGCCATGTCCTCCTCTGAGGATGAAGGCAGTCTGCGCTTTATTTA[T/A]GAGCTGCTGGGATGGGTWGAAGAAAMGCAAGATCTGCTGGAGCGAGCTGA.

For *Gt(macf1a–citrine)*^*ct68a*^ genotyping, we used For: ACGTAAACGGCCACAAGTTC, Rev: AAGTCGTGCTGCTTCATGTG. Denaturing: 94°C, 1:00 min. Annealing: 60°C, 45 sec Extension: 72°C, 30 sec X 30 cycles.

The *macf1a*^*p6cv/+*^ [5] and *macf1a*^*sa12708/+*^ heterozygotes exhibit a wild type phenotype and were used as controls alongside *macf1a*^*p6cv*^ [5] and *macf1a*^*sa12708*^ homozygous mutant alleles. In addition, both homozygous mutant alleles show indistinguishable mutant phenotypes, and we used them arbitrarily depending on fish availability.

### Fluorescence immunolabeling and RNA in situ hybridization

Ovaries were dissected from euthanized females and dissected carefully to preserve stage I-II oocytes and to remove later stage oocytes. Then the dissected ovaries were digested with 1.5 mg/ml collagenase I (Sigma-Aldrich) for 15 minutes in L-15 Medium (Sigma-Aldrich). Ovaries were fixed according to the acid fixation method [57] and kept overnight in 4% formaldehyde. Following washes in PBS, ovaries were kept in cold methanol at -20°C for at least 6 hours before use. For immunostaining, ovaries were washed and rehydrated in decreasing methanol percentages (75%, 50%, 25%) and washed finally in PBS 3 times (x). Ovaries were incubated in blocking solution containing PBS (0.3% Triton X-100, 1% BSA) for 1.5 to 2h. Primary antibodies were diluted in blocking solution and incubated overnight at 4°C. Ovaries were washed in blocking solution 4x15min and incubated with secondary antibodies in blocking solution for 90 min. Ovaries were washed in PBT (0.1% Triton) 4x15min and lastly incubated in PBT with DAPI (1:1000) and DiOC_6_ (1ug/ml) (Calbiochem) for 1–2 h. Then they were washed in PBT 4x10min, transferred into vectashield (Vector labs) and mounted for imaging. For Macf1 staining in Fig 3A–3C, after collagenase treatment we collected oocytes using a cell strainer (100 μm) (Fisherbrand^TM^), pipetted them into a 12-well plate, and performed the immunostaining in small volumes following the protocol described in [58]. This protocol for isolated oocytes may favor antibody penetration compared to the whole mount method used for Citrine staining and in Fig 8.

For CK detection, we used ovary tissue sections, where ovaries were first dissected and fixed in 4% formaldehyde overnight. After several washes in PBS, the ovaries were immersed in 30% sucrose for 48–72 hours to cryoprotect the samples, and stored in -20°C until cryosections were obtained. The immunostaining was performed on the mounted tissue sections.

#### Antibodies

The Buc antibody was made by YenZym (San Francisco, CA, USA). Buc epitope: residues 1–15 MEGINNNSQPMGVGQ were used to generate rabbit polyclonal antibodies as described [7]. Primary antibodies used were Buc (1:500), Macf1/ACF7 mouse (1:100 in Fig 3; 1:500 in Fig 8) (predicted peptide region 4184-4358aa in zebrafish Macf1a) [9], CK type II (1:50) (Progen) and GFP (1:500) (Life Technologies) for staining of *Gt(macf1a–citrine)*^*ct68a*^, Tg*(ef1a*:*dclk-GFP)* [31]*, Tg(bactin2:HsENSCONSIN17- 282-3xEGFP) [55]* ovaries. Secondary antibodies used were anti-rabbit IgG, or anti-mouse IgG1, Alexa 488, Alexa 594, Alexa 633 (all 1:1000, Molecular Probes).

#### In situ hybridization

Whole mount in situ hybridization was performed using the RNA-HCR method [27] following the company protocol (Molecular Instruments).

### Confocal microscopy and image processing

Images were acquired on a Zeiss LSM 710 confocal microscope using a 40X lens. The acquisition setting was set between samples and experiments to: XY resolution = 512x512 pixels, pinhole adjusted to 1.1μm of Z thickness, increments between stack images were 1μm, laser power and gain were set for each antibody. Acquired images were adjusted only in contrast/brightness. All figures were made using Adobe Photoshop and Illustrator CC 2014.

### Image quantification

#### Buc Bb/Buc Total ratio

We acquired confocal z-stacks encompassing the entire Buc signal and observed that Buc localized to the Bb showed greater signal intensity than Buc relocalizing to the cortex upon Bb disassembly. Using built-in filters in ImageJ software, we segmented the total Buc signal (Buc in the Bb and relocalized to the cortex) and separately the more intense Buc signal in the Bb only. Buc z-stack images were acquired as explained above and processed by subtracting background and applying a median filter (5 pixel radius). Then, we used ImageJ software to create a z-projection of the images. In these conditions, we applied the threshold check from the BioVoxxel toolbox and chose the Otsu and Intermode algorithms as the most accurate thresholds in segmenting total Buc and Buc in the Bb, respectively. Next, we measured the area segmented by each filter to calculate Buc Bb/Total Buc ratio. The oocyte area was calculated by segmenting the whole oocyte surface using the percentile algorithm in the DAPI channel. DAPI labels the follicle cell nuclei that surround the oocyte, and thus outline the oocyte, which was used to calculate the oocyte diameter. Due to the high signal to background ratio for Buc labeling, only when necessary were the thresholds adjusted manually.

### Cytokeratin puncta measurements

Cytokeratin puncta were quantified using a MATLAB (Version 8.2.0.701 R2013b 64-bit, MathWorks) script. All images were pre-processed to reduce noise and to separate each oocyte into a single image. Cytokeratin positive puncta were identified using a simple threshold. The oocytes were sectioned into three regions (cytoplasm, nucleus, and Balbiani body) according to the intensity of DiOC_6_ staining. Additionally, a region of just the perimeter of the cytoplasm was defined as any cytoplasm within one Balbiani-body-diameter of the cytoplasmic membrane. Puncta density was calculated as puncta per area for each region.

### Live imaging of whole ovaries and isolated oocytes

Ovaries were dissected from adult fish (3 to 12 months) in L-15 media (60% in Hanks solution with gentamycin (50μg/ml) (Gibco)) supplemented with FBS (10%) and insulin (15μg/ml) at 28°C. In the media, further dissection of the ovary was performed to isolate small pieces containing mainly stage I oocytes. The ovaries were placed in a glass bottom dish and embedded in low-melt agarose (0.5%) prepared in the media solution. The dish was filled with media solution containing MitoTracker (500 nM, Molecular Probes) for 2–3 hours, then the media was replaced once and ovaries kept in the media throughout the imaging.

### Nocodazole, Latrunculin A and cold treatment

Nocodazole (50μM) (EMD Milipore) and latrunculin A (Sigma-Aldrich) (20μg/ml) were used to destabilize MTs and actin, respectively. When live imaging began, ovaries had been exposed to the drugs in the incubation media for no longer than 15–20 min. The control group was treated with DMSO in the same conditions. We used Mitotracker staining to monitor oocyte health and viability during the treatment.

For analysis of LatA treatment in fixed samples, ovaries from the same fish were divided into Latrunculin A (Sigma-Aldrich) (20μg/ml) and DMSO treated groups with two replicates for each condition. We tested incubation times of 6, 12 and 20 hours. After the treatment, ovaries were fixed as above and stained for Buc. We measured the oocyte diameter and analyzed the effect of LatA treatment only in stage I oocytes.

For cold treatment, ovaries were dissected as explained previously; ovaries were kept in culture media, and divided in tubes placed in ice in a cold room (4°C) and controls kept at 28°C, both for 120 min. Then ovaries were fixed and processed for immunostaining.

### *macf1a* cDNA sequencing

To generate *macf1a* cDNA we used the Superscript First-cDNA synthesis system (Invitrogen). First, using the *macf1a* predicted ORF sequence (~25 kb) (NCBI: XP_001920094.1), primers were designed to amplify a large piece of *macf1a* cDNA (~19 kb) made from ovary RNA. Forward: CCACCGAAAAACAGGAGAACAC; Reverse: GCTCCACTTGAAACCTCTTCGC. Instead a ~6.5 kb product was obtained that was cloned into pCR-XL-TOPO (Invitrogen) (kindly provided by Tripti Gupta). We sequenced the 6.5 kb cDNA and found that it contained three regions from the *macf1a* predicted cDNA sequence: bp 3767–4482, 12314–15370 and 20453–23180. The 4482–12314 gap corresponds to exons 35–39; exon 35 contains the Macf1a PRD (~7 kb) domain. We amplified from ovary cDNA ~1.5 kb of exon 35 (NCBI:XP_001920094.1) using primers from exon 35 and flanking exon 34. We also amplified from ovary cDNA and sequenced exons 36–39, from flanking exon 35. In the ~25 kb predicted transcript (NCBI: XP_001920094.1), 29 spectrin repeats are predicted. We identified 2 spectrin repeats in exons 36–39, 14 spectrin repeats in the 6.5 kb cDNA spanning sequences 12314–15370, and another 13 were identified in cDNA by sequencing the gap in the 6.5 kb cDNA between 15370–20453 bp, corresponding to exons 57–75, for a total of 29 spectrin repeats. To complete the *macf1a* cDNA, we performed 5’ RACE, amplifying overlapping fragments upstream of 3955 bp of the 25 kb predicted transcript (NCBI:XP_001920094.1) to assemble a fragment containing the *macf1a* CH1-CH2 (ABD) and Plakin domains. Using 3’ RACE from the 23,180 bp position, we assembled a fragment containing the MTBD (~3 kb). See the list of primers in S1 Table.

### CRISPR/Cas9 genome editing

*macf1a* deletion mutants were created using CRIPSR-Cas9 mediated mutagenesis. The intron targets selected contained a PAM sequence for Cas9 targeting. The sgRNA constructs to target *macf1a* introns were purchased from the University of Utah Mutation Generation and Detection Core, which cloned them into plasmids containing an upstream T7 promoter and flanked by a DraI restriction site. To synthesize sgRNAs, we used the T7 MEGAshortscript kit (Ambion) and a clean-up step with MEGAclear kit (Ambion), following the manufacturer’s protocol.

For injections, we mixed sgRNAs (Table 1) and Cas9 protein (180–200 pg) (purchased from the University of Utah Mutation Generation and Detection Core for Crispr reagents) and injected 1.2–1.5 nl of the CRISPR mix into one-cell stage zebrafish embryos. At 24 to 48 hpf, a fraction (~25%) of the injected embryos were euthanized and DNA was extracted. We performed HRMA analysis on single embryos using MeltDoctor HRM Master Mix (Applied Biosystems) to determine the mutation rate, and PCR analysis to detect genomic deletions. When a high (~80–100%) mutation rate was obtained, the remaining injected embryos were raised to adulthood. We crossed F0 adults carrying *macf1a* ^*p1CH1*^ and *macf1a* ^*p2CH1CH2*^ deletions to wild type fish to produce F1 *macf1a* ^*p1CH1*^ and *macf1a* ^*p2CH1CH2*^ heterozygotes. We then crossed these heterozygotes to either a *macf1a*^*sa12708*^*/*+ female or a *macf1a*^*sa12708*^ homozygous male to obtain F2 transheterozygous ovaries of *macf1a* ^*p1CH1*^ /*macf1a*^*sa12708*^ or *macf1a* ^*p2CH1CH2*^ */macf1a*^*sa12708*^. In the next generation, we incrossed the F2 fish to obtain homozygous mutants for analysis.

HRMA primers:

Intron 3. Product: 120bp.
Forward (For) AACCTGTTGGTTCCATTTGAAGTAT
Reverse (Rev) GATTTGCTCAACCCCTTGCTCA
Intron 5. Product: 132bp
For TGCAGCAGACTGGAGATGAA
Rev GGATAGAGAGGAAGCCCGGA
Intron 8. Product: 127bp
For CCAGAGCAGAACAAACCCTA
Rev TGAACAAATCATTGCAGATG
Intron 34. Product: 109bp
For AGTCAGTTCCGGGCAGCATA
Rev ACACACTGATCGAGGTTTCGG
Intron 38. Product: 167bp
For GCATACGTGGACATACGTGA
Rev GTCCAGGTTCTGATTGGCTG

For PCR deletion analysis, we combined three primers to amplify the wild type and mutant alleles. PCR conditions:

Denaturing: 94°C, 1:00 min
Annealing: Primers 1) and 2) 59°C, 45 sec; 3) 58°C, 1 min.
Extension: 72°C, 45 sec
X 30 cycles.
  1) Deletion ABD (CH1) primers: wild type: 420bp; deletion: 520bp.
Intron 3 For TTCCACATCTGGGTTTGTGT
Intron 3 Rev TCTCAGGCTGAAACACATCTGA
Intron 5 Rev CTGGATGAGGACAGGAGGGA
  2) Deletion ABD (CH1-CH2) primers: wild type: 420 bp; deletion: 505 bp.
Intron 3 For TTCCACATCTGGGTTTGTGT
Intron 3 Rev TCTCAGGCTGAAACACATCTGA
Intron 8 Rev CATACAGCCTCTTCACCACTGT
  3) Deletion PRD primers: wild type: 420 bp; deletion: 505 bp.
Intron 34 For CTAACAGCTGCCGGGAGAAA
Intron 34 Rev ACACACTGATCGAGGTTTCGG
Intron 38 Rev AATAGTGCCTCTGCTCTGGC

### Statistical analysis

All statistical analysis and plotting was performed using the GraphPad Prism 6 and Excel software.

### MATLAB script for CK quantification

See S1 Methods.

## Supporting information

S1 Fig Latrunculin A treatment of stage I oocytes. Ovaries treated with DMSO (A-D) or LatA (E-H) for 6h or 12h, then fixed and stained with phalloidin (magenta) (A, B, E and F) or Buc (red) (C, D, G and H). Arrowheads point to Buc localized to the Bb and the cortex. After 6h of treatment, no effect was found on the Bb or nucleus in 18 DMSO or 22 LatA treated oocytes. After 12h of treatment, 19 DMSO-treated oocytes were normal, whereas 4/22 LatA-treated oocytes showed Buc cortical detachment, three of which showed an acentric nucleus. N ≥ 5 ovaries. Scale bar: 20 μm.
(TIF)

S2 Fig A) Detection of *macf1a*-ABD (CH1) and (CH1-CH2) deletions in the *macf1a* gene and cDNA. Scheme indicates intron targets for deleting *macf1a* ABD (CH1) and (CH1-CH2). Partial genomic sequence of introns 3 and 5 in WT and the genomic size between the selected CRISPR target sites is indicated. In green the predicted Cas9 cut site and the underlying gray line marks the joined sites of introns after Cas9 cutting and repair. Below is the cDNA sequence for *macf1a*^*p1CH1*^ and *macf1a*^*p2CH1-CH2*^. The exon composition (only the first few amino acids are shown) in WT compared to mutants confirms the intended exon deletions in *macf1a*^*p1CH1*^and *macf1a*^*p2CH1CH2*^. The primer locations for amplifying *macf1a* cDNA are indicated. B) Detection of *macf1a*-PRD domain deletion in the *macf1a* gene and cDNA. Scheme indicates intron targets for deleting the *macf1a*-PRD. Partial genomic sequence of introns 34 and 38 in WT indicating the genomic size between the selected CRISPR target sites, in green the predicted Cas9 cut site, and the underlying gray line marks the joined sites of introns 34 and 38 after Cas9 cutting and repair. Below is the deleted genomic DNA and cDNA sequence for *macf1a*^*p*3*PRD*^. The primer locations for amplifying *macf1a* cDNA are indicated. C) PCR products from ovary cDNA amplifying *macf1a* ABD and PRD. Primer combinations and expected PCR product sizes are shown along with the bands detected in WT and mutants (lanes 1–3, ABD; 4–9, PRD). Arrowheads (black and red) indicate the mutant bands at the expected size and asterisks indicate the WT bands.
(TIF)

S1 Table List of primers used to sequence *macf1a* ovary cDNA. (DOCX)

S1 Methods Matlab script for cytokeratin quantification in Fig 5. (RTF)

S1 Movie Live imaging of control *Tg(ef1a*:*dclk-GFP)* ovaries in which Dclk-GFP decorates MTs in green. Control ovaries are incubated in DMSO.
(AVI)

S2 Movie Live imaging of control ovaries stained with Mitotracker (red) to label the Bb. Control ovaries are incubated in DMSO. The arrow points to the Bb.
(AVI)

S3 Movie Live imaging of *Tg(ef1a*:*dclk-GFP)* ovaries in which Dclk-GFP decorates MTs in green. Ovary was treated with nocodazole (50 μM) for 140 min, which effectively depolymerizes MTs.
(AVI)

S4 Movie Live imaging of ovaries stained with Mitotracker (red) to label the Bb. The nocodazole (50 μM) treatment for 140 min caused no defect in the Bb or nuclear positioning. The arrow points to the Bb.
(AVI)

## References

[1] Boke E, Ruer M, Wuhr M, Coughlin M, Lemaitre R, Gygi SP, et al Amyloid-like Self-Assembly of a Cellular Compartment. Cell. 2016;166(3):637–50. doi: 10.1016/j.cell.2016.06.051 . 27471966 10.1016/j.cell.2016.06.051 PMC5082712

[2] Marlow FL, Mullins MC. Bucky ball functions in Balbiani body assembly and animal-vegetal polarity in the oocyte and follicle cell layer in zebrafish. Dev Biol. 2008;321(1):40–50. Epub 2008/06/28. doi: 10.1016/j.ydbio.2008.05.557 ; PubMed Central PMCID: PMC2606906. 18582455 10.1016/j.ydbio.2008.05.557 PMC2606906

[3] Bontems F, Stein A, Marlow F, Lyautey J, Gupta T, Mullins MC, et al Bucky ball organizes germ plasm assembly in zebrafish. Curr Biol. 2009;19(5):414–22. Epub 2009/03/03. doi: 10.1016/j.cub.2009.01.038 . 19249209 10.1016/j.cub.2009.01.038

[4] Escobar-Aguirre M, Elkouby YM, Mullins MC. Localization in Oogenesis of Maternal Regulators of Embryonic Development In: Pelegri F, Danilchik M, Sutherland A, editors. Vertebrate Development: Maternal to Zygotic Control. Gewerbestrasse 11, 6330 Cham, Switzerland: Springer International Publishing; 2016 p. 173–208.

[5] Gupta T, Marlow FL, Ferriola D, Mackiewicz K, Dapprich J, Monos D, et al Microtubule actin crosslinking factor 1 regulates the Balbiani body and animal-vegetal polarity of the zebrafish oocyte. Plos Genet. 2010;6(8):e1001073 Epub 2010/09/03. doi: 10.1371/journal.pgen.1001073 ; PubMed Central PMCID: PMC2924321. 20808893 10.1371/journal.pgen.1001073 PMC2924321

[6] Dosch R, Wagner DS, Mintzer KA, Runke G, Wiemelt AP, Mullins MC. Maternal control of vertebrate development before the midblastula transition: mutants from the zebrafish I. Dev Cell. 2004;6(6):771–80. Epub 2004/06/05. doi: 10.1016/j.devcel.2004.05.002 . 15177026 10.1016/j.devcel.2004.05.002

[7] Heim AE, Hartung O, Rothhamel S, Ferreira E, Jenny A, Marlow FL. Oocyte polarity requires a Bucky ball-dependent feedback amplification loop. Development. 2014;141(4):842–54. doi: 10.1242/dev.090449 ; PubMed Central PMCID: PMC3912829. 24496621 10.1242/dev.090449 PMC3912829

[8] Chang P, Torres J, Lewis RA, Mowry KL, Houliston E, King ML. Localization of RNAs to the mitochondrial cloud in Xenopus oocytes through entrapment and association with endoplasmic reticulum. Mol Biol Cell. 2004;15(10):4669–81. Epub 2004/08/05. doi: 10.1091/mbc.E04-03-0265 ; PubMed Central PMCID: PMC519158. 15292452 10.1091/mbc.E04-03-0265 PMC519158

[9] Karakesisoglou I, Yang Y, Fuchs E. An epidermal plakin that integrates actin and microtubule networks at cellular junctions. J Cell Biol. 2000;149(1):195–208. Epub 2000/04/04. ; PubMed Central PMCID: PMC2175090. 10747097 10.1083/jcb.149.1.195 PMC2175090

[10] Kodama A, Karakesisoglou I, Wong E, Vaezi A, Fuchs E. ACF7: an essential integrator of microtubule dynamics. Cell. 2003;115(3):343–54. . 14636561 10.1016/s0092-8674(03)00813-4

[11] Lin CM, Chen HJ, Leung CL, Parry DA, Liem RK. Microtubule actin crosslinking factor 1b: a novel plakin that localizes to the Golgi complex. J Cell Sci. 2005;118(Pt 16):3727–38. Epub 2005/08/04. doi: 10.1242/jcs.02510 . 16076900 10.1242/jcs.02510

[12] Chen HJ, Lin CM, Lin CS, Perez-Olle R, Leung CL, Liem RK. The role of microtubule actin cross-linking factor 1 (MACF1) in the Wnt signaling pathway. Genes Dev. 2006;20(14):1933–45. doi: 10.1101/gad.1411206 ; PubMed Central PMCID: PMC1522081. 16815997 10.1101/gad.1411206 PMC1522081

[13] Wu X, Kodama A, Fuchs E. ACF7 regulates cytoskeletal-focal adhesion dynamics and migration and has ATPase activity. Cell. 2008;135(1):137–48. doi: 10.1016/j.cell.2008.07.045 ; PubMed Central PMCID: PMC2703712. 18854161 10.1016/j.cell.2008.07.045 PMC2703712

[14] Antonellis PJ, Pollock LM, Chou SW, Hassan A, Geng R, Chen X, et al ACF7 is a hair-bundle antecedent, positioned to integrate cuticular plate actin and somatic tubulin. J Neurosci. 2014;34(1):305–12. doi: 10.1523/JNEUROSCI.1880-13.2014 ; PubMed Central PMCID: PMC3866489. 24381291 10.1523/JNEUROSCI.1880-13.2014 PMC3866489

[15] Lee S, Harris KL, Whitington PM, Kolodziej PA. short stop is allelic to kakapo, and encodes rod-like cytoskeletal-associated proteins required for axon extension. J Neurosci. 2000;20(3):1096–108. Epub 2000/01/29. . 10648715 10.1523/JNEUROSCI.20-03-01096.2000 PMC6774164

[16] Lee S, Kolodziej PA. The plakin Short Stop and the RhoA GTPase are required for E-cadherin-dependent apical surface remodeling during tracheal tube fusion. Development. 2002;129(6):1509–20. Epub 2002/03/07. . 11880359 10.1242/dev.129.6.1509

[17] Lee S, Kolodziej PA. Short Stop provides an essential link between F-actin and microtubules during axon extension. Development. 2002;129(5):1195–204. . 11874915 10.1242/dev.129.5.1195

[18] Subramanian A, Prokop A, Yamamoto M, Sugimura K, Uemura T, Betschinger J, et al Shortstop recruits EB1/APC1 and promotes microtubule assembly at the muscle-tendon junction. Curr Biol. 2003;13(13):1086–95. Epub 2003/07/05. . 12842007 10.1016/s0960-9822(03)00416-0

[19] Roper K, Brown NH. A spectraplakin is enriched on the fusome and organizes microtubules during oocyte specification in Drosophila. Curr Biol. 2004;14(2):99–110. Epub 2004/01/24. . 14738730

[20] Sanchez-Soriano N, Travis M, Dajas-Bailador F, Goncalves-Pimentel C, Whitmarsh AJ, Prokop A. Mouse ACF7 and drosophila short stop modulate filopodia formation and microtubule organisation during neuronal growth. J Cell Sci. 2009;122(Pt 14):2534–42. Epub 2009/07/03. doi: 10.1242/jcs.046268 ; PubMed Central PMCID: PMC2704885. 19571116 10.1242/jcs.046268 PMC2704885

[21] Bottenberg W, Sanchez-Soriano N, Alves-Silva J, Hahn I, Mende M, Prokop A. Context-specific requirements of functional domains of the Spectraplakin Short stop in vivo. Mech Dev. 2009;126(7):489–502. Epub 2009/05/05. doi: 10.1016/j.mod.2009.04.004 . 19409984 10.1016/j.mod.2009.04.004

[22] Bosher JM, Hahn BS, Legouis R, Sookhareea S, Weimer RM, Gansmuller A, et al The Caenorhabditis elegans vab-10 spectraplakin isoforms protect the epidermis against internal and external forces. J Cell Biol. 2003;161(4):757–68. Epub 2003/05/21. doi: 10.1083/jcb.200302151 ; PubMed Central PMCID: PMC2199363. 12756232 10.1083/jcb.200302151 PMC2199363

[23] Kim HS, Murakami R, Quintin S, Mori M, Ohkura K, Tamai KK, et al VAB-10 spectraplakin acts in cell and nuclear migration in Caenorhabditis elegans. Development. 2011;138(18):4013–23. Epub 2011/08/13. doi: 10.1242/dev.059568 ; PubMed Central PMCID: PMC3160096. 21831923 10.1242/dev.059568 PMC3160096

[24] Messitt TJ, Gagnon JA, Kreiling JA, Pratt CA, Yoon YJ, Mowry KL. Multiple kinesin motors coordinate cytoplasmic RNA transport on a subpopulation of microtubules in Xenopus oocytes. Dev Cell. 2008;15(3):426–36. Epub 2008/09/06. doi: 10.1016/j.devcel.2008.06.014 ; PubMed Central PMCID: PMC2581415. 18771961 10.1016/j.devcel.2008.06.014 PMC2581415

[25] Gard DL, Cha BJ, King E. The organization and animal-vegetal asymmetry of cytokeratin filaments in stage VI Xenopus oocytes is dependent upon F-actin and microtubules. Dev Biol. 1997;184(1):95–114. Epub 1997/04/01. doi: 10.1006/dbio.1997.8508 . 9142987 10.1006/dbio.1997.8508

[26] Selman K, Wallace RA, Sarka A, Qi XP. STAGES OF OOCYTE DEVELOPMENT IN THE ZEBRAFISH, BRACHYDANIO-RERIO. Journal of Morphology. 1993;218(2):203–24. doi: 10.1002/jmor.1052180209 10.1002/jmor.1052180209 29865471

[27] Choi HM, Chang JY, Trinh le A, Padilla JE, Fraser SE, Pierce NA. Programmable in situ amplification for multiplexed imaging of mRNA expression. Nat Biotechnol. 2010;28(11):1208–12. doi: 10.1038/nbt.1692 ; PubMed Central PMCID: PMCPMC3058322. 21037591 10.1038/nbt.1692 PMC3058322

[28] Ge X, Grotjahn D, Welch E, Lyman-Gingerich J, Holguin C, Dimitrova E, et al Hecate/Grip2a acts to reorganize the cytoskeleton in the symmetry-breaking event of embryonic axis induction. Plos Genet. 2014;10(6):e1004422 Epub 2014/06/27. doi: 10.1371/journal.pgen.1004422 ; PubMed Central PMCID: PMC4072529. 24967891 10.1371/journal.pgen.1004422 PMC4072529

[29] Trinh le A, Hochgreb T, Graham M, Wu D, Ruf-Zamojski F, Jayasena CS, et al A versatile gene trap to visualize and interrogate the function of the vertebrate proteome. Genes Dev. 2011;25(21):2306–20. doi: 10.1101/gad.174037.111 ; PubMed Central PMCID: PMC3219234. 22056673 10.1101/gad.174037.111 PMC3219234

[30] Behrndt M, Salbreux G, Campinho P, Hauschild R, Oswald F, Roensch J, et al Forces driving epithelial spreading in zebrafish gastrulation. Science. 2012;338(6104):257–60. doi: 10.1126/science.1224143 . 23066079 10.1126/science.1224143

[31] Tran LD, Hino H, Quach H, Lim S, Shindo A, Mimori-Kiyosue Y, et al Dynamic microtubules at the vegetal cortex predict the embryonic axis in zebrafish. Development. 2012;139(19):3644–52. doi: 10.1242/dev.082362 . 22949618 10.1242/dev.082362

[32] Gard DL. Organization, nucleation, and acetylation of microtubules in Xenopus laevis oocytes: a study by confocal immunofluorescence microscopy. Dev Biol. 1991;143(2):346–62. Epub 1991/02/01. . 1991557 10.1016/0012-1606(91)90085-h

[33] Dahlem TJ, Hoshijima K, Jurynec MJ, Gunther D, Starker CG, Locke AS, et al Simple methods for generating and detecting locus-specific mutations induced with TALENs in the zebrafish genome. Plos Genet. 2012;8(8):e1002861 doi: 10.1371/journal.pgen.1002861 ; PubMed Central PMCID: PMCPMC3420959. 22916025 10.1371/journal.pgen.1002861 PMC3420959

[34] Bernier G, Mathieu M, De Repentigny Y, Vidal SM, Kothary R. Cloning and characterization of mouse ACF7, a novel member of the dystonin subfamily of actin binding proteins. Genomics. 1996;38(1):19–29. . 8954775 10.1006/geno.1996.0587

[35] Alves-Silva J, Sanchez-Soriano N, Beaven R, Klein M, Parkin J, Millard TH, et al Spectraplakins promote microtubule-mediated axonal growth by functioning as structural microtubule-associated proteins and EB1-dependent +TIPs (tip interacting proteins). J Neurosci. 2012;32(27):9143–58. Epub 2012/07/06. doi: 10.1523/JNEUROSCI.0416-12.2012 . 22764224 10.1523/JNEUROSCI.0416-12.2012 PMC3666083

[36] Wang JT, Smith J, Chen BC, Schmidt H, Rasoloson D, Paix A, et al Regulation of RNA granule dynamics by phosphorylation of serine-rich, intrinsically disordered proteins in C. elegans. eLife. 2014;3:e04591 doi: 10.7554/eLife.04591 ; PubMed Central PMCID: PMC4296509. 25535836 10.7554/eLife.04591 PMC4296509

[37] Brangwynne CP, Eckmann CR, Courson DS, Rybarska A, Hoege C, Gharakhani J, et al Germline P granules are liquid droplets that localize by controlled dissolution/condensation. Science. 2009;324(5935):1729–32. doi: 10.1126/science.1172046 . 19460965 10.1126/science.1172046

[38] Leung CL, Sun D, Zheng M, Knowles DR, Liem RK. Microtubule actin cross-linking factor (MACF): a hybrid of dystonin and dystrophin that can interact with the actin and microtubule cytoskeletons. J Cell Biol. 1999;147(6):1275–86. Epub 1999/12/22. ; PubMed Central PMCID: PMC2168091. 10601340 10.1083/jcb.147.6.1275 PMC2168091

[39] Nashchekin D, Fernandes AR, St Johnston D. Patronin/Shot Cortical Foci Assemble the Noncentrosomal Microtubule Array that Specifies the Drosophila Anterior-Posterior Axis. Dev Cell. 2016;38(1):61–72. doi: 10.1016/j.devcel.2016.06.010 ; PubMed Central PMCID: PMCPMC4943857. 27404359 10.1016/j.devcel.2016.06.010 PMC4943857

[40] Elkouby YM, Jamieson-Lucy A, Mullins MC. Oocyte Polarization Is Coupled to the Chromosomal Bouquet, a Conserved Polarized Nuclear Configuration in Meiosis. Plos Biol. 2016;14(1):e1002335 Epub 2016/01/08. doi: 10.1371/journal.pbio.1002335 . 26741740 10.1371/journal.pbio.1002335 PMC4704784

[41] Gard DL, Affleck D, Error BM. Microtubule organization, acetylation, and nucleation in Xenopus laevis oocytes: II. A developmental transition in microtubule organization during early diplotene. Dev Biol. 1995;168(1):189–201. Epub 1995/03/01. doi: 10.1006/dbio.1995.1071 . 7883073 10.1006/dbio.1995.1071

[42] Kloc M, Etkin LD. Two distinct pathways for the localization of RNAs at the vegetal cortex in Xenopus oocytes. Development. 1995;121(2):287–97. . 7539356 10.1242/dev.121.2.287

[43] Wuhr M, Guttler T, Peshkin L, McAlister GC, Sonnett M, Ishihara K, et al The Nuclear Proteome of a Vertebrate. Curr Biol. 2015;25(20):2663–71. doi: 10.1016/j.cub.2015.08.047 ; PubMed Central PMCID: PMCPMC4618192. 26441354 10.1016/j.cub.2015.08.047 PMC4618192

[44] Laporte C, Vetter G, Loudes AM, Robinson DG, Hillmer S, Stussi-Garaud C, et al Involvement of the secretory pathway and the cytoskeleton in intracellular targeting and tubule assembly of Grapevine fanleaf virus movement protein in tobacco BY-2 cells. Plant Cell. 2003;15(9):2058–75. Epub 2003/09/04. doi: 10.1105/tpc.013896 ; PubMed Central PMCID: PMC181331. 12953111 10.1105/tpc.013896 PMC181331

[45] Sosa BA, Rothballer A, Kutay U, Schwartz TU. LINC complexes form by binding of three KASH peptides to domain interfaces of trimeric SUN proteins. Cell. 2012;149(5):1035–47. Epub 2012/05/29. doi: 10.1016/j.cell.2012.03.046 ; PubMed Central PMCID: PMC3383001. 22632968 10.1016/j.cell.2012.03.046 PMC3383001

[46] Crisp M, Liu Q, Roux K, Rattner JB, Shanahan C, Burke B, et al Coupling of the nucleus and cytoplasm: role of the LINC complex. J Cell Biol. 2006;172(1):41–53. Epub 2005/12/29. doi: 10.1083/jcb.200509124 ; PubMed Central PMCID: PMC2063530. 16380439 10.1083/jcb.200509124 PMC2063530

[47] Meinke P, Nguyen TD, Wehnert MS. The LINC complex and human disease. Biochem Soc Trans. 2011;39(6):1693–7. Epub 2011/11/23. doi: 10.1042/BST20110658 . 22103509 10.1042/BST20110658

[48] Link J, Leubner M, Schmitt J, Gob E, Benavente R, Jeang KT, et al Analysis of meiosis in SUN1 deficient mice reveals a distinct role of SUN2 in mammalian meiotic LINC complex formation and function. Plos Genet. 2014;10(2):e1004099 Epub 2014/03/04. doi: 10.1371/journal.pgen.1004099 ; PubMed Central PMCID: PMC3937131. 24586178 10.1371/journal.pgen.1004099 PMC3937131

[49] Lombardi ML, Lammerding J. Keeping the LINC: the importance of nucleocytoskeletal coupling in intracellular force transmission and cellular function. Biochem Soc Trans. 2011;39(6):1729–34. Epub 2011/11/23. doi: 10.1042/BST20110686 . 22103516 10.1042/BST20110686 PMC4589539

[50] Brosig M, Ferralli J, Gelman L, Chiquet M, Chiquet-Ehrismann R. Interfering with the connection between the nucleus and the cytoskeleton affects nuclear rotation, mechanotransduction and myogenesis. Int J Biochem Cell Biol. 2010;42(10):1717–28. Epub 2010/07/14. doi: 10.1016/j.biocel.2010.07.001 . 20621196 10.1016/j.biocel.2010.07.001

[51] Guo L, Degenstein L, Dowling J, Yu QC, Wollmann R, Perman B, et al Gene targeting of BPAG1: abnormalities in mechanical strength and cell migration in stratified epithelia and neurologic degeneration. Cell. 1995;81(2):233–43. . 7736575 10.1016/0092-8674(95)90333-x

[52] Koenig M, Hoffman EP, Bertelson CJ, Monaco AP, Feener C, Kunkel LM. Complete cloning of the Duchenne muscular dystrophy (DMD) cDNA and preliminary genomic organization of the DMD gene in normal and affected individuals. Cell. 1987;50(3):509–17. . 3607877 10.1016/0092-8674(87)90504-6

[53] Bouameur JE, Favre B, Borradori L. Plakins, a versatile family of cytolinkers: roles in skin integrity and in human diseases. J Invest Dermatol. 2014;134(4):885–94. doi: 10.1038/jid.2013.498 . 24352042 10.1038/jid.2013.498

[54] Jorgensen LH, Mosbech MB, Faergeman NJ, Graakjaer J, Jacobsen SV, Schroder HD. Duplication in the microtubule-actin cross-linking factor 1 gene causes a novel neuromuscular condition. Sci Rep. 2014;4:5180 doi: 10.1038/srep05180 ; PubMed Central PMCID: PMCPMC4046130. 24899269 10.1038/srep05180 PMC4046130

[55] Wuhr M, Obholzer ND, Megason SG, Detrich HW 3rd, Mitchison TJ. Live imaging of the cytoskeleton in early cleavage-stage zebrafish embryos. Methods Cell Biol. 2011;101:1–18. doi: 10.1016/B978-0-12-387036-0.00001-3 . 21550437 10.1016/B978-0-12-387036-0.00001-3 PMC6551615

[56] Smith SM, Maughan PJ. SNP genotyping using KASPar assays. Methods Mol Biol. 2015;1245:243–56. doi: 10.1007/978-1-4939-1966-6_18 . 25373762 10.1007/978-1-4939-1966-6_18

[57] Fernandez J, Fuentes R. Fixation/permeabilization: new alternative procedure for immunofluorescence and mRNA in situ hybridization of vertebrate and invertebrate embryos. Dev Dyn. 2013;242(5):503–17. doi: 10.1002/dvdy.23943 . 23389988 10.1002/dvdy.23943

[58] Anger M, Stein P, Schultz RM. CDC6 requirement for spindle formation during maturation of mouse oocytes. Biol Reprod. 2005;72(1):188–94. Epub 2004/09/24. doi: 10.1095/biolreprod.104.035451 . 15385409 10.1095/biolreprod.104.035451

