# Supplementary figures and images for: Microtubule-actin crosslinking factor 1 (Macf1) domain function in Balbiani body dissociation and nuclear positioning
Source: PLoS Genet. 2017 Sep 7;13(9):e1006983. doi: 10.1371/journal.pgen.1006983 (PMC5605089; doi:10.1371/journal.pgen.1006983)

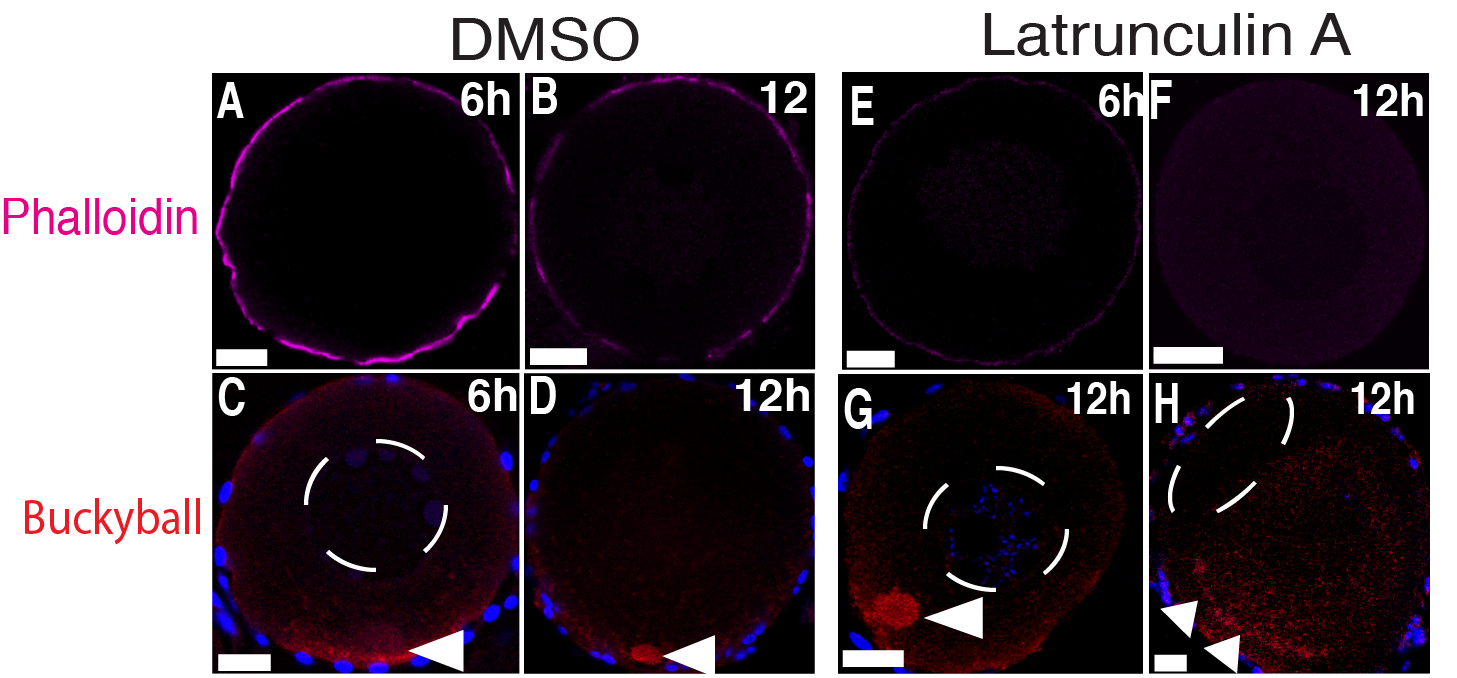

Supplement: S1 Fig — Ovaries treated with DMSO (A-D) or LatA (E-H) for 6h or 12h, then fixed and stained with phalloidin (magenta) (A, B, E and F) or Buc (red) (C, D, G and H). Arrowheads point to Buc localized to the Bb and the cortex. After 6h of treatment, no effect was found on the Bb or nucleus in 18 DMSO or 22 LatA treated oocytes. After 12h of treatment, 19 DMSO-treated oocytes were normal, whereas 4/22 LatA-treated oocytes showed Buc cortical detachment, three of which showed an acentric nucleus. N ≥ 5 ovaries. Scale bar: 20 μm. (TIF) [file pgen.1006983.s001.tif]

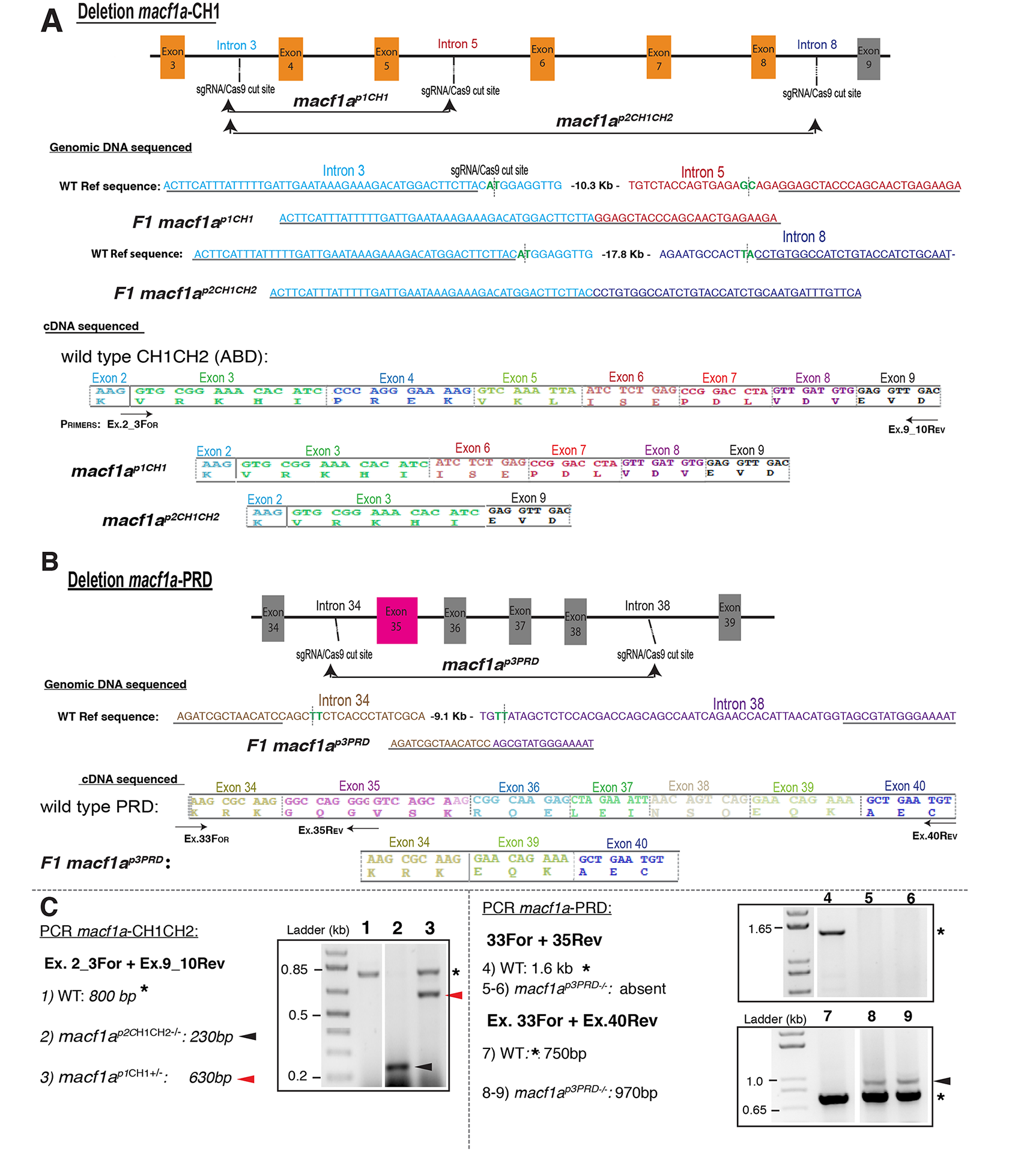

Supplement: S2 Fig — A) Detection of macf1a-ABD (CH1) and (CH1-CH2) deletions in the macf1a gene and cDNA. Scheme indicates intron targets for deleting macf1a ABD (CH1) and (CH1-CH2). Partial genomic sequence of introns 3 and 5 in WT and the genomic size between the selected CRISPR target sites is indicated. In green the predicted Cas9 cut site and the underlying gray line marks the joined sites of introns after Cas9 cutting and repair. Below is the cDNA sequence for macf1ap1CH1 and macf1ap2CH1-CH2. The exon composition (only the first few amino acids are shown) in WT compared to mutants confirms the intended exon deletions in macf1ap1CH1and macf1ap2CH1CH2. The primer locations for amplifying macf1a cDNA are indicated. B) Detection of macf1a-PRD domain deletion in the macf1a gene and cDNA. Scheme indicates intron targets for deleting the macf1a-PRD. Partial genomic sequence of introns 34 and 38 in WT indicating the genomic size between the selected CRISPR target sites, in green the predicted Cas9 cut site, and the underlying gray line marks the joined sites of introns 34 and 38 after Cas9 cutting and repair. Below is the deleted genomic DNA and cDNA sequence for macf1ap3PRD. The primer locations for amplifying macf1a cDNA are indicated. C) PCR products from ovary cDNA amplifying macf1a ABD and PRD. Primer combinations and expected PCR product sizes are shown along with the bands detected in WT and mutants (lanes 1–3, ABD; 4–9, PRD). Arrowheads (black and red) indicate the mutant bands at the expected size and asterisks indicate the WT bands. (TIF) [file pgen.1006983.s002.tif]
