# Supplementary material for: Microtubule-actin crosslinking factor 1 (Macf1) domain function in Balbiani body dissociation and nuclear positioning
Source: PLoS Genet. 2017 Sep 7;13(9):e1006983. doi: 10.1371/journal.pgen.1006983 (PMC5605089; doi:10.1371/journal.pgen.1006983)
Supplement: S1 Table — (DOCX) [file pgen.1006983.s003.docx]

Supplementary Table 1. List of *macf1a* cDNA primers

| **NCBI: XP_001920094.1. (start position)** | **Sequence** |
| --- | --- |
| 691 | GCTGAAGATGTTGATGTGCCG |
| 1455 | CAGGAAGGGTCATTTCTCTACGG |
| 1886 | GCAAACTGCTGGAACATTCGTC |
| 2633 | TGCCATCTGTGTGTTTCACCG |
| 2645 | TTAGGGGGAGGAACGGTGAAAC |
| 3050 | AGAGCATCAAGTCTCACCTGGAGG |
| 3388 | TTCTGACCTGACTTTCTGCCGC |
| 3767 | CCACCGAAAAACAGGAGAACAC |
| 3788 | TGTCAGTTTGTTCGGGCTGTG |
| 3878 | AACTGGATGAGTGCCAGACC |
| 4103 | GTGATGCGCTACGAAGACTGG |
| 4231 | GATGTGCGACAAGTGATGGCTC |
| 4550 | CACAGCATGGCCTCATAGAC |
| 4613 | GTCAGGCTGGAAAAGACCAC |
| 5234 | CTGTTCAGCTTGGCCTGATG |
| 5282 | CCCTGTTGTTAGCTGTCGTG |
| 5691 | ATCTGTCAGTTCTTCGTCACCCCC |
| 5842 | CGCTTAGTCCTGCTAAAACCAG |
| 5894 | CTGGTATGGCAAGGTTTTTTGAGC |
| 5904 | GTTCAACTGGTATGGCAAGG |
| 6586 | TCTGGGTCCTTCAAAGATGC |
| 7764 | GGGGGAAACTACCAGCGATAAG |
| 7764 | GGGGGAAACTACCAGCGATAAG |
| 8357 | CCTGTAAAGGCTCTGTCAATGCTC |
| 8357 | CCTGTAAAGGCTCTGTCAATGCTC |
| 10679 | CTATGCGTCACTCAGCCAATAATG |
| 11565 | GCGAACCTCAATGTGTCTCACC |
| 11565 | GGTGAGACACATTGAGGTTCGC |
| 11565 | GCGAACCTCAATGTGTCTCACC |
| 12181 | GCATCATTGAGACCAACGGC |
| 14194 | AGTGTGGATGACTACAGGCAGACC |
| 14488 | TTAGGTTGGGCTCTTTGGCAGG |
| 14488 | TTAGGTTGGGCTCTTTGGCAGG |
| 16214 | GGATTGCGGAATGTGAGGTAAAG |
| 16592 | CGCTCCAGGCTTTTGTTGCTAC |
| 16801 | CAGTGCTGTTCATCATTGGAGTCC |
| 16931 | GCCTGTGAAGCCAAAGAGTCAG |
| 17546 | TTGGCTATCAGTTCCTCTGTGTCAC |
| 18513 | CAATCAATGGCTGGATAGAGTGG |
| 19014 | GAGAGACGCACTGAAGGTGAAGAC |
| 19167 | CTGGGTAATCAGAGTCTCAGTTTCG |
| 19590 | AACGATGGCAGGAGTTTGTCC |
| 20481 | TCACACTCACGCCACACTGGATA |
| 21053 | CCAAAATGGAAGACAGAAGGGG |
| 21089 | AAACCTGTCGCCAGTGAAACGG |
| 21588 | GGTTTATGACACCACAGTGCGTTC |
| 21746 | AAACTGACCAGAGAACAGGAGGG |
| 22269 | ACACAGCGAGAATGGCTTCG |
| 22271 | AAGCCATTCTCGCTGTGTGC |
| 22704 | TGGTAATCCTCGCCTCACTCAG |
| 22861 | GACTTCTTGTGGTTCATCCAACG |
| 23158 | GCTCCACTTGAAACCTCTTCGC |
| 24880 | GGTGCTTCCGTCACGAAAGAAC |

.
