# Supplementary material for: Microtubule-actin crosslinking factor 1 (Macf1) domain function in Balbiani body dissociation and nuclear positioning
Source: PLoS Genet. 2017 Sep 7;13(9):e1006983. doi: 10.1371/journal.pgen.1006983 (PMC5605089; doi:10.1371/journal.pgen.1006983)
Supplement: S1 Methods — (RTF) [file pgen.1006983.s004.rtf]

Script to quantify cytokeratin puncta density within oocytes


clc, clear, close all

load('oocyte ref.mat')
load('previousthresh.mat')

titles = {'Bb Puncta Density', 'Perimiter Puncta Density',...
    'All Cytoplasm Puncta Density', 'Pixels per um', 'Source Pathname',...
    'Source Filename', 'Slice', 'Output Pathname', 'Output Filename',...
    'Perimiter Radius', 'Bb User Defined?', 'Bb ID Threshold',...
    'Nucleus ID Threshold', 'Cytoplasm ID Threshold',...
    'Puncta ID Threshold 1', 'Puncta ID Threshold 2'};
data1 = titles;
guess4 = 80;
guess5 = 0;

%%
for jj = 1:82
    
filenum = jj;
% load the file of interest
load(['/Users/jamiesonlucy/Desktop/matias matlab analysis/tifs/oocyte slices/oocyte-'...
    num2str(filenum) '.mat'])

% get the resolution of each individual file
% load up the table with all the file info
% get the resolution out of the metadata
tiffilename = [oocytereference{filenum+1,1}, oocytereference{filenum+1,2}];
imginf = imfinfo(tiffilename);
pixperum = imginf(1).XResolution;


% pull the slices apart!
dapi = oocyteslice(:,:,1);
dioc6 = oocyteslice(:,:,2);
cyto = oocyteslice(:,:,3);
logfield = logical(oocyteslice(:,:,4));

% crop!
dapi(~logfield) = NaN;
dioc6(~logfield) = NaN;
cyto(~logfield) = NaN;

% balbiani body threshold guess
guess1 = prevrun{jj+1, 12};
% user input to ID the balbiani body
[bbim, bbthresh, bbuserinput] = userbbinput(dioc6, dapi, guess1);
bbtrue = sum(sum(bbim)) > 0;

% nucleus threshold guess
guess2 = prevrun{jj+1, 13};
% user input to ID the nucleus
[nucim, nucthresh, nucuserinput] = usernucinput(dioc6, dapi, guess2);

% cytoplasm/cortex threshold guess
guess3 = prevrun{jj+1, 14};
% user input to ID the cortex
[corim, corthresh, coruserinput] = usercytoinput(dioc6, dapi, guess3);

%%
pathname = '/Users/jamiesonlucy/Desktop/matias matlab analysis/tifs/oocyte slices copy/';
oocyteslice(:,:,5) = bbim;
oocyteslice(:,:,6) = nucim;
oocyteslice(:,:,7) = corim;

filenum = num2str(jj);
save(['oocyte-' filenum '.mat'], 'oocyteslice')

end

%%

[thresh1, thresh2, localmax, allPuncta] = cytofiddler(cyto, guess4, guess5);
guess4 = thresh1;
guess5 = thresh2;
 
%%
cyto2 = imhmax(cyto, imhmaxthresh);
disk = fspecial('disk', 3);
cytoblur = imfilter(cyto2, disk);
cytoblur(~(cytoblur > cytothresh)) = 0;
localmax = imregionalmax(cytoblur);

% Find the locations of all the maxima and convert them into (x,y) format
stats = regionprops(localmax, 'PixelList');
allPuncta = zeros(numel(stats), 2);
for kk = 1:numel(stats)
     Px = stats(kk).PixelList;
    allPuncta(kk,:) = Px(1,:);
end
 
%% section image and bin the puncta

if bbtrue
    bbstats = regionprops(bbim, 'MajorAxisLength', 'MinorAxisLength');
    rad = floor((bbstats(1).MajorAxisLength+bbstats(1).MinorAxisLength)/2);
else
    rad = 40;
end
eroder = strel('disk',rad);
middle = imerode(corim, eroder);


% define points overlapping with those objects
if bbtrue
    bbpts = localmax & bbim;
    corpts = localmax & (corim & ~bbim & ~nucim);
    perimpts = localmax & (corim & ~bbim & ~middle & ~nucim);
else
    corpts = localmax & corim & ~nucim;
    perimpts = localmax & ~middle & ~nucim;
end
middlepts = localmax & middle;
% 
% turn the points into x,y values for plotting
if bbtrue
    stats = regionprops(bbpts, 'PixelList');
    bbPuncta = zeros(numel(stats), 2);
    for kk = 1:numel(stats)
        Px = stats(kk).PixelList;
        bbPuncta(kk,:) = Px(1,:);
    end
end
% 
stats = regionprops(corpts, 'PixelList');
corPuncta = zeros(numel(stats), 2);
for kk = 1:numel(stats)
    Px = stats(kk).PixelList;
    corPuncta(kk,:) = Px(1,:);
end

stats = regionprops(perimpts, 'PixelList');
perimPuncta = zeros(numel(stats), 2);
for kk = 1:numel(stats)
    Px = stats(kk).PixelList;
    perimPuncta(kk,:) = Px(1,:);
end

stats = regionprops(middlepts, 'PixelList');
middlePuncta = zeros(numel(stats), 2);
for kk = 1:numel(stats)
    Px = stats(kk).PixelList;
    middlePuncta(kk,:) = Px(1,:);
end

%%  plot the stuff to check that it looks nice
hold off
figure
hold on
imagesc(dioc6)
map = [0, 0, 0; 0, 0.1, 0; 0, 0.2, 0; 0, 0.3, 0; 0, 0.4, 0; 0, 0.5, 0;...
    0, 0.6, 0; 0, 0.7, 0; 0, 0.8, 0; 0, 0.9, 0; 0, 1, 0];
colormap(map)
plot(allPuncta(:,1), allPuncta(:,2), 'w.', 'MarkerSize', 20);
plot(corPuncta(:,1), corPuncta(:,2), 'r.');
if bbtrue
    plot(bbPuncta(:,1), bbPuncta(:,2), 'k.');
end
plot(perimPuncta(:,1), perimPuncta(:,2), 'g.');

%% get puncta densities
% count the points
if bbtrue
    propbb = bwconncomp(bbpts);
end
propcor = bwconncomp(corpts);
propperim = bwconncomp(perimpts);
propmiddle = bwconncomp(middlepts);

if bbtrue
    numbb = propbb.NumObjects;
end
numcor = propcor.NumObjects;
numperim = propperim.NumObjects;
nummiddle = propmiddle.NumObjects;

% calculate the area
if bbtrue
    bbarea = sum(sum(double(bbim)));
corarea = sum(sum(double(corim & ~bbim & ~nucim)));
perimarea = sum(sum(double(corim & ~bbim & ~middle & ~nucim)));
end
middlearea = sum(sum(double(middle)));

if bbtrue
    bbdensity = numbb/bbarea;
else
    bbdensity = NaN;
end
cordensity = numcor/corarea;
perimdensity = numperim/perimarea;
middledensity = nummiddle/middlearea;

waitfor(gcf)

lineOdata = {bbdensity, perimdensity, cordensity, pixperum,...
    oocytereference{filenum+1,:}, rad, bbuserinput, bbthresh, nucthresh,...
    corthresh, thresh1, thresh2};
data1 = [data1; lineOdata];

end

%% Saving parameters for excel

prompt = 'Filename (no extension)';
dlg_title = 'Save CSV';
num_lines = 1;
response = inputdlg(prompt,dlg_title,num_lines);
responsestr = response{1,1};
cell2csv([responsestr,'.csv'], data1, ',', '2011')

%% Saving parameters for MATLAB

data1 = prevrun;
save('previousthresh.mat', 'prevrun')


Custom functions within puncta quantification script (user interfaces)


function [bbid, bbthresh, userinput] = userbbinput(dioc6, dapi, guess)

%initialize stuff
fig1 = figure('Position', [50, 150, 1000, 500]);
axes1 = axes('Position', [0.05 0.15 0.4 0.8]);
axes2 = axes('Position', [0.55 0.15 0.4 0.8]);

% show initial thresholding guess
bbim = dioc6 > guess;
bbim = bwareaopen(bbim, 60);
imshow(bbim, 'Parent', axes1)

% make empty balbiani body thing
bbfield = zeros(size(dioc6,1), size(dioc6, 2));

% plot initial picture of oocyte slice
rgb(:,:,2) = dioc6;
rgb(:,:,1) = bbfield;
rgb(:,:,3) = dapi;
imshow(rgb, [0, 256], 'Parent', axes2)

% make slider
sld = uicontrol('Style', 'slider',...
    'Min',0,'Max',256,'Value',guess,...
    'Position', [400 20 120 20],...
    'Callback', @changethresh);

% make the brush size chooser
sz = uicontrol('Style', 'edit', 'String', '10',...
    'Position', [180 20, 50, 20],...
    'Callback', @changesz);

% make the clear button
btn1 = uicontrol('Style', 'pushbutton', 'String', 'Clear',...
    'Position', [20 20 50 20],...
    'Callback', @clearit);

% make the okay button
btn2 = uicontrol('Style', 'pushbutton', 'String', 'Done',...
    'Position', [300 20 50 20],...
    'Callback', @okaythen);

% default not clicking
drag = 0;

% choose size of brush
rad = str2num(get(sz, 'String'));

% gonna need a field to act on to check for circles
[X, Y] = meshgrid(1:size(dioc6,2), 1:size(dioc6,1));

% set the clicking up
set(gcf, 'WindowButtonDownFcn',@draw,...
    'WindowButtonUpFcn',@stopdrawing, 'WindowButtonMotionFcn',@keepdrawing,...
    'KeyPressFcn', @arrowarrow);

waitfor(fig1)

%slider callback
    function changethresh(~,~)
        val =  get(sld, 'Value');
        bbim = dioc6 > val;
        bbim = bwareaopen(bbim, 60);
        imshow(bbim, 'Parent', axes1);
    end

% brush size function
    function changesz(varargin)
        rad = str2num(get(sz, 'String'));
    end

% clear button fuction
    function clearit(varargin)
        bbfield(:,:) = 0;
        rgb(:,:,2) = dioc6;
        rgb(:,:,1) = double(bbfield)*256;
        rgb(:,:,3) = dapi;
        imshow(rgb, [0, 256], 'Parent', axes2)
        drawnow
    end

% okay button function
    function okaythen(varargin)
        % generate output
% balbiani body ID
bbid = bbim | bbfield;
bbid = imfill(bbid, 'holes');
% what's the threshold?
bbthresh = get(sld, 'Value');
% was there user input?  1 = yes, 0 = no
userinput = sum(sum(bbfield)) > 0;

        close(fig1)
    end

% click mouse
    function draw(varargin)
        % tell computer dragging has started
        drag = 1;
        % get the position
        posit = get(gca, 'CurrentPoint');
        positx = posit(1,1);
        posity = posit(1,2);
        
        % find a circle around the position of the click
        drawcirc = ((X - positx).^2 + (Y - posity).^2) < rad^2;
        % add that circle to the output thing
        bbfield = bbfield | drawcirc;

        % display
        rgb(:,:,2) = dioc6;
        rgb(:,:,1) = double(bbfield)*256;
        rgb(:,:,3) = dapi;
        imshow(rgb, [0, 256], 'Parent', axes2)
        drawnow
    end

% move mouse
    function keepdrawing(varargin)
        % if dragging, do the same thing you do on a click for every point
        % you drag through
        if drag == 1
            posit = get(gca, 'CurrentPoint');
            positx = posit(1,1);
            posity = posit(1,2);
            drawcirc = ((X - positx).^2 + (Y - posity).^2) < rad^2;
            bbfield = bbfield | drawcirc;

            rgb(:,:,2) = dioc6;
            rgb(:,:,1) = double(bbfield)*256;
            rgb(:,:,3) = dapi;
            imshow(rgb, [0, 256], 'Parent', axes2)
            drawnow
        end
    end

% lift mouse
    function stopdrawing(varargin)
        % stop dragging, aka unclick
        drag = 0;
    end

end


function [cytoid, cytothresh, userinput] = usercytoinput(dioc6, dapi, guess)

%initialize stuff
fig1 = figure('Position', [50, 150, 1000, 500]);
axes1 = axes('Position', [0.05 0.15 0.4 0.8]);
axes2 = axes('Position', [0.55 0.15 0.4 0.8]);

% show initial thresholding guess
cytoim = dioc6 > guess;
%calculate area for each labeled object
stats = regionprops(cytoim,'Area');
maxarea = max([stats.Area]);
cytoim = bwareaopen(cytoim, (maxarea-1));
imshow(cytoim, 'Parent', axes1)

% make empty balbiani body thing
cytofield = zeros(size(dioc6,1), size(dioc6, 2));

% plot initial picture of oocyte slice
rgb(:,:,2) = dioc6;
rgb(:,:,1) = cytofield;
rgb(:,:,3) = dapi;
imshow(rgb, [0, 256], 'Parent', axes2)

% make slider
sld = uicontrol('Style', 'slider',...
    'Min',0,'Max',256,'Value',guess,...
    'Position', [400 20 120 20],...
    'Callback', @changethresh);

% make the brush size chooser
sz = uicontrol('Style', 'edit', 'String', '10',...
    'Position', [180 20, 50, 20],...
    'Callback', @changesz);

% make the clear button
btn1 = uicontrol('Style', 'pushbutton', 'String', 'Clear',...
    'Position', [20 20 50 20],...
    'Callback', @clearit);

% make the okay button
btn2 = uicontrol('Style', 'pushbutton', 'String', 'Done',...
    'Position', [300 20 50 20],...
    'Callback', @okaythen);

% default not clicking
drag = 0;

% choose size of brush (possibly later a user input on the figure)
rad = str2num(get(sz, 'String'));

% gonna need a field to act on to check for circles
[X, Y] = meshgrid(1:size(dioc6,2), 1:size(dioc6,1));

% set the clicking up
set(gcf, 'WindowButtonDownFcn',@draw,...
    'WindowButtonUpFcn',@stopdrawing, 'WindowButtonMotionFcn',@keepdrawing,...
    'KeyPressFcn', @arrowarrow);

waitfor(fig1)

%slider callback
    function changethresh(~,~)
        val =  get(sld, 'Value');
        cytoim = dioc6 > val;
        %calculate area for each labeled object
        stats = regionprops(cytoim,'Area');
        maxarea = max([stats.Area]);
        cytoim = bwareaopen(cytoim, (maxarea-1));
        imshow(cytoim, 'Parent', axes1)
    end

% brush size function
    function changesz(varargin)
        rad = str2num(get(sz, 'String'));
    end

% clear button fuction
    function clearit(varargin)
        cytofield(:,:) = 0;
        rgb(:,:,2) = dioc6;
        rgb(:,:,1) = double(cytofield)*256;
        rgb(:,:,3) = dapi;
        imshow(rgb, [0, 256], 'Parent', axes2)
        drawnow
    end

% okay button function
    function okaythen(varargin)
        % generate output
        % balbiani body ID
        cytoid = cytoim | cytofield;
        cytoid = imfill(cytoid, 'holes');
        % what's the threshold?
        cytothresh = get(sld, 'Value');
        % was there user input?  1 = yes, 0 = no
        userinput = sum(sum(cytofield)) > 0;
        
        close(fig1)
    end

% click mouse
    function draw(varargin)
        % tell computer dragging has started
        drag = 1;
        % get the position
        posit = get(gca, 'CurrentPoint');
        positx = posit(1,1);
        posity = posit(1,2);
        
        % find a circle around the position of the click
        drawcirc = ((X - positx).^2 + (Y - posity).^2) < rad^2;
        % add that circle to the output thing
        cytofield = cytofield | drawcirc;
        
        % display
        rgb(:,:,2) = dioc6;
        rgb(:,:,1) = double(cytofield)*256;
        rgb(:,:,3) = dapi;
        imshow(rgb, [0, 256], 'Parent', axes2)
        drawnow
    end

% move mouse
    function keepdrawing(varargin)
        % if dragging, do the same thing you do on a click for every point
        % you drag through
        if drag == 1
            posit = get(gca, 'CurrentPoint');
            positx = posit(1,1);
            posity = posit(1,2);
            drawcirc = ((X - positx).^2 + (Y - posity).^2) < rad^2;
            cytofield = cytofield | drawcirc;
            
            rgb(:,:,2) = dioc6;
            rgb(:,:,1) = double(cytofield)*256;
            rgb(:,:,3) = dapi;
            imshow(rgb, [0, 256], 'Parent', axes2)
            drawnow
        end
    end

% lift mouse
    function stopdrawing(varargin)
        % stop dragging, aka unclick
        drag = 0;
    end

end


function [nucid, nucthresh, userinput] = usernucinput(dioc6, dapi, guess)

%initialize stuff
fig1 = figure('Position', [50, 150, 1000, 500]);
axes1 = axes('Position', [0.05 0.15 0.4 0.8]);
axes2 = axes('Position', [0.55 0.15 0.4 0.8]);

% show initial thresholding guess
nucimg = dioc6 > guess;
nucfilled = imfill(nucimg, 'holes');
nucmessy = nucfilled & ~nucimg;
nucfinal = bwareaopen(nucmessy, 1500);
imshow(nucfinal, 'Parent', axes1)

% make empty balbiani body thing
nucfield = zeros(size(dioc6,1), size(dioc6, 2));

% plot initial picture of oocyte slice
rgb(:,:,2) = dioc6;
rgb(:,:,1) = nucfield;
rgb(:,:,3) = dapi;
imshow(rgb, [0, 256], 'Parent', axes2)

% make slider
sld = uicontrol('Style', 'slider',...
    'Min',0,'Max',256,'Value',guess,...
    'Position', [400 20 120 20],...
    'Callback', @changethresh);

% make the brush size chooser
sz = uicontrol('Style', 'edit', 'String', '10',...
    'Position', [180 20, 50, 20],...
    'Callback', @changesz);

% make the clear button
btn1 = uicontrol('Style', 'pushbutton', 'String', 'Clear',...
    'Position', [20 20 50 20],...
    'Callback', @clearit);

% make the okay button
btn2 = uicontrol('Style', 'pushbutton', 'String', 'Done',...
    'Position', [300 20 50 20],...
    'Callback', @okaythen);

% default not clicking
drag = 0;

% choose size of brush (possibly later a user input on the figure)
rad = str2num(get(sz, 'String'));

% gonna need a field to act on to check for circles
[X, Y] = meshgrid(1:size(dioc6,2), 1:size(dioc6,1));

% set the clicking up
set(gcf, 'WindowButtonDownFcn',@draw,...
    'WindowButtonUpFcn',@stopdrawing, 'WindowButtonMotionFcn',@keepdrawing,...
    'KeyPressFcn', @arrowarrow);

waitfor(fig1)

%slider callback
    function changethresh(~,~)
        val =  get(sld, 'Value');
        nucimg = dioc6 > val;
        nucfilled = imfill(nucimg, 'holes');
        nucmessy = nucfilled & ~nucimg;
        nucfinal = bwareaopen(nucmessy, 600);
        imshow(nucfinal, 'Parent', axes1)
        
        imshow(nucfinal, 'Parent', axes1);
    end

% brush size function
    function changesz(varargin)
        rad = str2num(get(sz, 'String'));
    end

% clear button fuction
    function clearit(varargin)
        nucfield(:,:) = 0;
        rgb(:,:,2) = dioc6;
        rgb(:,:,1) = double(nucfield)*256;
        rgb(:,:,3) = dapi;
        imshow(rgb, [0, 256], 'Parent', axes2)
        drawnow
    end

% okay button function
    function okaythen(varargin)
        % generate output
        % balbiani body ID
        nucid = nucfinal | nucfield;
        nucid = imfill(nucid, 'holes');
        % what's the threshold?
        nucthresh = get(sld, 'Value');
        % was there user input?  1 = yes, 0 = no
        userinput = sum(sum(nucfield)) > 0;
        close(fig1)
    end

% click mouse
    function draw(varargin)
        % tell computer dragging has started
        drag = 1;
        % get the position
        posit = get(gca, 'CurrentPoint');
        positx = posit(1,1);
        posity = posit(1,2);
        
        % find a circle around the position of the click
        drawcirc = ((X - positx).^2 + (Y - posity).^2) < rad^2;
        % add that circle to the output thing
        nucfield = nucfield | drawcirc;
        
        % display
        rgb(:,:,2) = dioc6;
        rgb(:,:,1) = double(nucfield)*256;
        rgb(:,:,3) = dapi;
        imshow(rgb, [0, 256], 'Parent', axes2)
        drawnow
    end

% move mouse
    function keepdrawing(varargin)
        % if dragging, do the same thing you do on a click for every point
        % you drag through
        if drag == 1
            posit = get(gca, 'CurrentPoint');
            positx = posit(1,1);
            posity = posit(1,2);
            drawcirc = ((X - positx).^2 + (Y - posity).^2) < rad^2;
            nucfield = nucfield | drawcirc;
            
            rgb(:,:,2) = dioc6;
            rgb(:,:,1) = double(nucfield)*256;
            rgb(:,:,3) = dapi;
            imshow(rgb, [0, 256], 'Parent', axes2)
            drawnow
        end
    end

% lift mouse
    function stopdrawing(varargin)
        % stop dragging, aka unclick
        drag = 0;
    end

end
